# Colour-sensitive conjugated polymer inkjet-printed pixelated artificial retina model studied via a bio-hybrid photovoltaic device

**DOI:** 10.1038/s41598-020-77819-z

**Published:** 2020-12-08

**Authors:** Manuela Ciocca, Pavlos Giannakou, Paolo Mariani, Lucio Cinà, Aldo Di Carlo, Mehmet O. Tas, Hiroki Asari, Serena Marcozzi, Antonella Camaioni, Maxim Shkunov, Thomas M. Brown

**Affiliations:** 1 Department of Electronic Engineering, University of Rome Tor Vergata, Via del Politecnico 1, Rome, Italy; 2 Department of Electrical and Electronic Engineering, Faculty of Engineering and Physical Sciences, Advanced Technology Institute, University of Surrey, Guildford, UK; 3 Cicci Research Srl., Via Giordania 227, Grosseto, Italy; 4 Istituto di Struttura della Materia, CNR-ISM, via Fosso del Cavaliere 100, Rome, Italy; 5 Epigenetics and Neurobiology Unit, European Molecular Biology Laboratory, Via Ramarini 32, Monterotondo, Italy; 6 Department of Biomedicine and Prevention, University of Rome Tor Vergata, Via Montpellier 1, Rome, Italy

**Keywords:** Biomedical engineering, Biomaterials, Biomaterials, Electrical and electronic engineering, Electronics, photonics and device physics

## Abstract

In recent years, organic electronic materials have been shown to be a promising tool, even transplanted in vivo, for transducing light stimuli to non-functioning retinas. Here we developed a bio-hybrid optoelectronic device consisting of patterned organic polymer semiconductors interfaced with an electrolyte solution in a closed sandwich architecture in order to study the photo-response of photosensitive semiconducting layers or patterns in an environment imitating biological extracellular fluids. We demonstrate an artificial retina model composed of on an array of 42,100 pixels made of three different conjugated polymers via inkjet printing with 110 pixels/mm^2^ packing density. Photo-sensing through three-colour pixelation allows to resolve incoming light spectrally and spatially. The compact colour sensitive optoelectronic device represents an easy-to-handle photosensitive platform for the study of the photo response of artificial retina systems.

## Introduction

According to the World Health Organization around 285 million people worldwide are visually impaired, of whom 39 million are blind^1^. Retinal degenerative illness such as Retinitis Pigmentosa (RP) and Age-related Macular Degeneration (AMD) are the leading causes of partial or total vision loss. All retinal degenerative diseases present a damage to photoreceptor cells. Photoreceptors are light-sensitive specialized neurons found in the retina, the nervous tissue covering the inside-back of the eyeball. Human retina contains two kinds of photoreceptors, rods and cones, responsible for scotopic monochromatic and photopic trichromatic vision respectively (S-cones, M-cones and L-cones, often labelled as “blue”, “green” and “red” cones sensing short, mid and long wavelengths respectively). Photoreceptors convert light into electrical signals that travel along the optical nerve to the brain where vision is perceived. Without a proper functionality of photoreceptors, vision is principally compromised because photons are not converted into electrical triggers needed to change the resting membrane potentials of retinal cells to let the visual process begin.

With the development of biomedical engineering in the last decades, retinal prostheses, designed to partially restore vision, have seen progress^2,3^. These electronic devices are mainly based on silicon or metallic and rigid electrodes^4^ and possess poor flexibility and biocompatibility^5^. Moreover they often require external power supplies, video camera and cables^6^. Alternatively, nowadays the fast-growing field of bio-electronics based on organic semiconductors is offering a novel scenario for biomedical devices^7,8^. Organic semiconductors can be interfaced with biological matter ranging from small biomolecules such as DNA^9–11^ to cells and tissues^5^. In the last few years an exciting avenue of research has consisted in interfacing degenerated retinas with semiconducting polymers to elicit a response in the former upon photoexcitation of the latter by transduction of signals^12–15^. The effectiveness of interfacing conjugated polymers or quantum dots with retinas have been demonstrated even in vivo in blind rats either via implantation^12,13^ or injection^16,17^. The flexible nature of organic electronic materials provides better bio-mechanical compatibility, making them suitable as biomedical implants. Undoubtedly their ability to conduct ions, in addition to electrons and holes, opens a new communication channel with biology due to the importance of ion fluxes in biological systems^18^. Furthermore, these materials in an engineered nanoscale, including carbon nanotubes^19,20^ graphene^21,22^ and conjugated polymers^23,24^, have the potential to interact with biological systems on a molecular scale, offering unprecedented levels of control over physiological activity^25,26^.

Poly-3-hexylthiophene (P3HT) has been the polymer of choice in the vast majority of these studies^27,28^ being used as optical mediator in biological in vitro and in vivo applications for retinal prosthetic devices^29^. Moreover, the synthesis of conjugated polymers with different spectral response enables absorption of light at different frequencies and this is a crucial feature for future multi-chromatic retinal prostheses^30,31^. Up to today, to the best of our knowledge, the colour response of these materials was only studied with a few types of living cells^32,33^. In addition, the spectral responsivity was considered individually for one polymer at a time while never for different polymers together in the same device. Furthermore, all these studies employed standard electrophysiological tools such as patch-clamps amplifiers and Multi-Electrode Arrays (MEAs) for their analysis, where one of the electrodes is a reference electrode immersed in a bath of electrolyte solution with a flat/electrode/polymer system^2,3,12,13,25–27,32,33^.

Here we present a novel closed type sandwich structure device consisting of two transparent electrodes separated by a spacer encapsulation layer which encloses a semiconducting photosensitive film over the bottom electrode interfaced with electrolyte imitating extracellular fluid. Furthermore, rather than using conventional microfabrication methods (e.g. spin coating and photolithographic patterning), in this work conjugated polymers were pixelated using inkjet printing technology, exploiting one of the main advantages of these types of materials, which is patterned additive printing, in order to prepare a simplified concentric model of a spectrally-sensitive retina which enables to investigate the geometrical and spectral response of the pixelated structure immersed in an biological electrolyte. The demonstration of a spectrally sensitive photo-response of these systems, depending locally on the absorption spectra of deposited polymer pixels, represents an important step towards a colour sensitive artificial retina.

## Results

The results section is organized following our research approach: first we designed and validated a bio-hybrid sandwich structure consisting of a polymer semiconductor and a physiological electrolyte solution; second we developed a simplified concentric model of a pixelated retina via inkjet printing of different conjugated polymers that mimic the three spectra of rods and two cones typical of mammalian retinas.

### Bio-hybrid device: design, fabrication and biocompatibility

**Figure 1:**
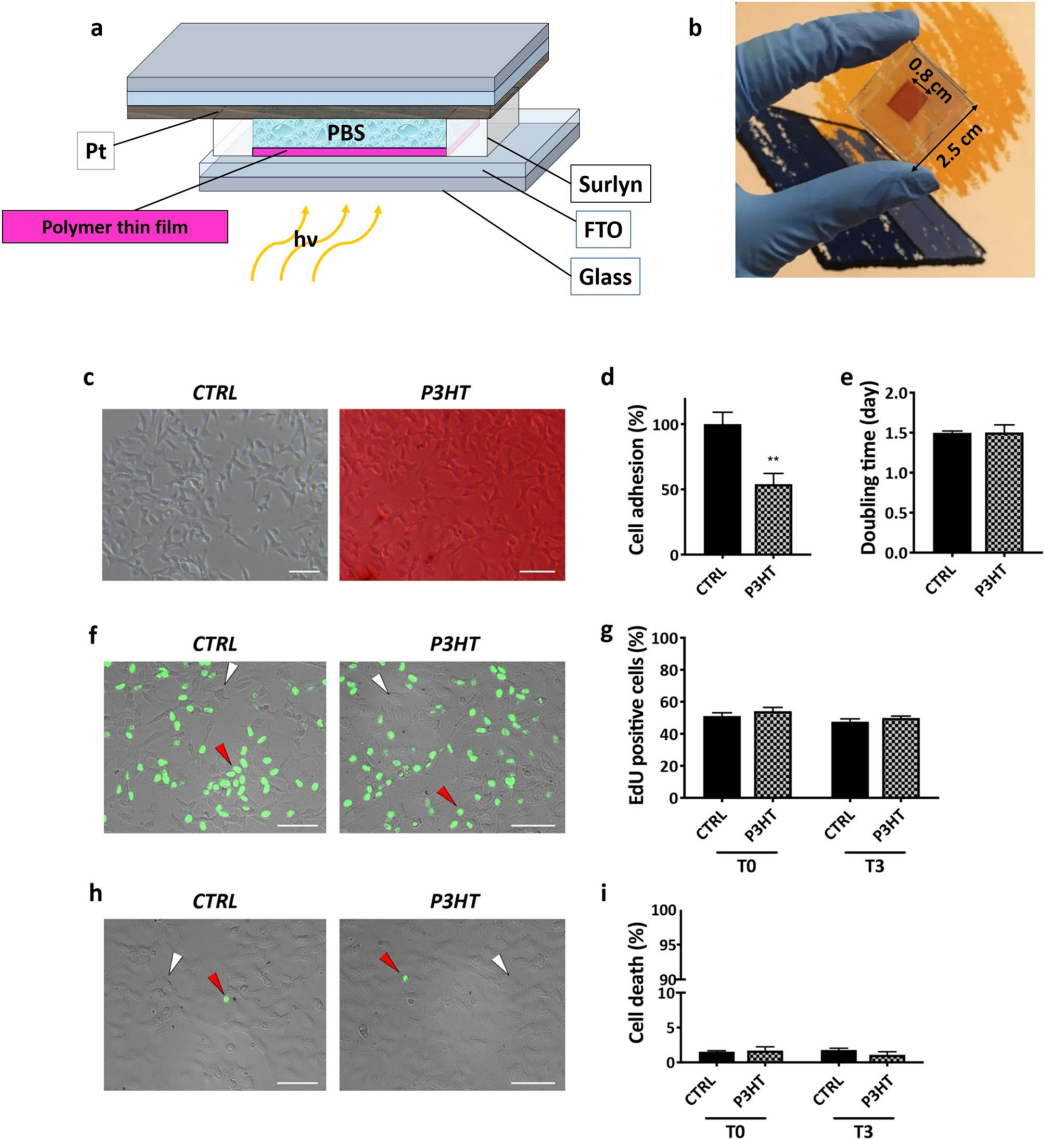
Bio-hybrid device layout for biological system/organic electronic interfaces and biocompatibility evaluated as cell adhesion, proliferation and viability. (**a**) Schematic of the device. It consists of two transparent conductive electrodes. The photo-electrode (polymer thin film on FTO) is grounded. The platinum layer deposited on FTO is the counter-electrode. A phosphate buffered saline solution (PBS) was used as electrolyte and it is enclosed in the inner chamber (60 µm thick) formed between the two electrodes (Image created by using Microsoft PowerPoint). (**b**) Picture of the fabricated device. Dimensions are also shown. (**c**) Phase contrast micrographs of randomly selected fields of SH-SY5Y cells cultured on standard polystyrene dish (CTRL) and on Glass|FTO|P3HT substrate (P3HT) for 3 days. Scale bar = 50 μm. (**d**) Cell adhesion evaluated at 16 h after seeding SH-SY5Y cells on polystyrene dish (CTRL) and on Glass|FTO|P3HT (P3HT). Data are expressed as percentage of the CTRL. Statistical difference vs CTRL **p < 0.01 (Data were analysed with GraphPad Prism software V7.0). (**e**) Cell proliferation evaluated after three days of culture and expressed as cell doubling time for SH-SY5Y cultured on polystyrene dish (CTRL) and on the P3HT layer (Image J software 1.49 V was used. Data were analysed with GraphPad Prism software V7.0). (**f**) Representative micrographs of SH-SY5Y cells cultured on polystyrene dish (CTRL) and on Glass|FTO|P3HT substrate (P3HT) and analysed via the Click-iT EdU proliferation assay after 3 days of culture (T3). White and red arrowheads indicate non-proliferating and proliferating (EdU positive) cells, respectively. Scale bar = 50 μm. (**g**) Quantification of cell proliferation measured by Click-iT EdU proliferation assay, expressed as percentage of positive cells on the total number of cells analysed at the beginning (T0) and at the end (T3) of culture (Data were analysed with GraphPad Prism software V7.0). (**h**) Representative micrographs of SH-SY5Y cells cultured on polystyrene dish (CTRL) and on Glass|FTO|P3HT substrate (P3HT) and analysed via the in-situ Cell Viability Imaging assay after three days of culture (T3). White and red arrowheads indicate live and dead (green) cells, respectively. Scale bar = 50 μm. (**i**) Quantification of cell death measured by in-situ Cell Viability Imaging assay, expressed as percentage of dead (green) cells on the total number of cells analysed at the beginning (T0) and at the end (T3) of culture (Image J software 1.49 V was used).

Here we present a bio-hybrid device composed of both biological (physiological electrolyte solution mimicking extracellular fluid) and non-biological components. The developed device architecture consists of two transparent electrodes separated by a thermoplastic encapsulant with the photosensitive polymer thin film interfaced with an electrolyte mediator. The electrolyte solution was a buffered isotonic solution (Phosphate buffered saline solution, PBS) commonly employed in a biological context to reproduce a physiological extracellular fluid^34,35^. The concept of the device is the result of the amalgamation of different set-ups from different fields, i.e. those from electrophysiology^12,13,15^, organic photovoltaics (OPV)^9,36^ and Dye-Sensitized Solar Cells (DSSC)^37–39^. Figure 1a,b illustrates a schematic of the device structure. The working electrode (sensitive photo-electrode) is 100 nm-thick polymer semiconductor film, P3HT at first (see “Methods” section), spin-coated on a glass substrate covered with a transparent conducting oxide (fluorine tin oxide—FTO). P3HT is a prototype material for OPV applications^40^, it works as an absorber and electron donor material. The counter-electrode is a platinum (Pt) layer screen printed through a precursor on FTO-coated substrate and then fired at 480 °C for 30 min^41^. The latter procedure, borrowed from DSSC technology^42^, converts the used Pt precursor paste into catalytic Pt nanometre-sized islands that permit a facile exchange of electrons/ions at the electrode/electrolyte interface maintaining a high degree of transparency with a resulting transmittance of up to 79% between 400 and 700 nm^43^ (Supplementary Fig. S1). Moreover platinum electrodes are widely used in bioelectronic applications because they can safely deliver charge densities ranging from 0.15 to 5.57 mC/cm^2^^44^. PBS is contained in an inner chamber delimited by the thermoplastic sealant gasket (i.e. Surlyn). The presence of the biological electrolyte is important to provide a similar physiological aqueous environment for cells, mimicking eye conditions, and to test whether the polymer layer will retain its optoelectronic features in such biological/aqueous environment. The spacing between the two electrodes can be controlled via the thickness of the thermoplastic spacer. Here 60 µm thick gaskets were used representing a thickness which elicit measurable signals and potentially being able to accommodate smaller biological cells. Thicknesses greater than 60 µm can be used to place larger retinal tissues into the device for future studies. To address the issue of biocompatibility, human neuroblastoma cells SH-SY5Y were seeded on a P3HT layer and grown for up to 3 days, as described in “Methods” section. Cell adhesion, proliferation and viability were evaluated and compared with parallel cultures carried out with SH-SY5Y cells grown on tissue culture tested polystyrene dishes, as positive control (CTRL) (Fig. 1c). Adhesion was analysed 16 h after cell seeding (T0) and the results showed an adhesion rate of 50–60% on P3HT layer in comparison to control (CTRL = 100 ± 9.15%; P3HT = 54.07 ± 8.14%) (Fig. 1d). For the analysis of cell proliferation, the number of cells seeded on P3HT was adjusted based on the calculated rate of adhesion to have the same number of adhered cells at the beginning of culture (T0) in CTRL and P3HT conditions. When the adhered cells (CTRL = 1.49 ± 0.38 × 10^4^ cells/cm^2^; P3HT = 1.40 ± 0.29 × 10^4^ cells/cm^2^) were allowed to grow for 3 days, no significant differences were observed between the two culture conditions in the proliferation rate expressed as cell doubling time (CTRL = 1.50 ± 0.02 day; P3HT = 1.50 ± 0.10 day) (Fig. 1e). Proliferation was also confirmed by the Click-iT EdU proliferation assay performed on the cells at the beginning (T0) and after 3 days (T3) of culture (Fig. 1f,g). Indeed, data indicated comparable percentages of EdU positive, proliferating cells both at T0 and T3 on either substrates (T0: CTRL = 51.14 ± 2.06%; P3HT = 54.18 ± 2.37%; T3: CTRL = 47.58 ± 1.80%; P3HT = 49.98 ± 1.12%). Finally, cell death percentage analysed at T0 and T3 by the in-situ Cell Viability Imaging assay showed no differences between CTRL and P3HT (Fig. 1h,i). These results indicated no toxicity of the P3HT layer.

### Bio-hybrid device: opto-electronic characterization

**Figure 2:**
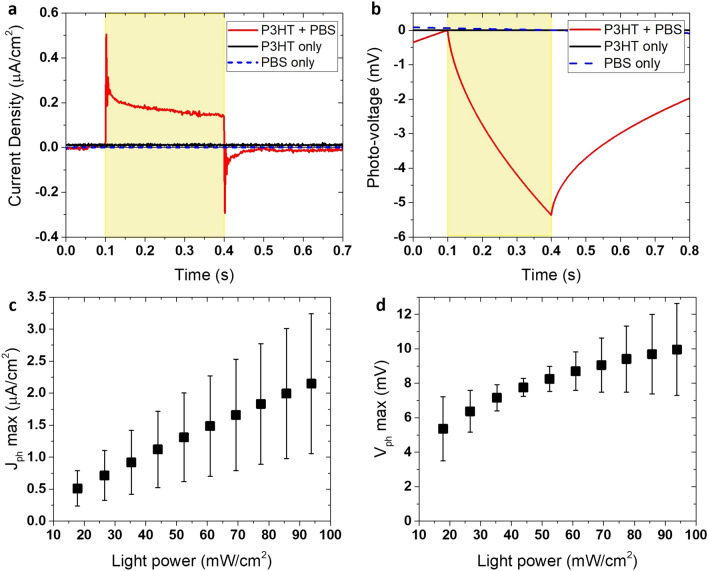
Bio-hybrid device optoelectrical characterization. (**a**) Transient current density generated by 300 ms white light stimulus (17.8 mW/cm^2^). (**b**) Transient photo-voltage recorded from the device under 300 ms white light (17.8 mW/cm^2^) stimulus. (**a**,**b**) black line: device containing only polymer thin film; dashed blue line: device containing only electrolyte solution; red line: device containing polymer (P3HT) and biological electrolyte (PBS). The shaded yellow area represents the duration of the light stimulus (300 ms). (**c**) Current capacitive peaks (J_ph_ max) show a linear dependence with the light power intensity. Plot is referred to J_ph_ first positive peaks. J_ph_ values are the mean on samples n = 3. Y-error bar is also shown. (**d**) Photo-Voltage as function of increasing light intensities. V_ph_ max values (in absolute value) are referred to the value reached after 300 ms from the light switched ON. Radiant intensities used for J_ph_ and V_ph_: 17.8, 26.6, 35.3, 43.9, 52.4, 60.9, 69.3, 77.4, 85.7, and 93.8 mW/cm^2^. (OriginPro 2016 was used).

White light (17.8 mW/cm^2^, standard cool white light spectrum Supplementary Fig. S2) was shone on three different device architectures containing: (i) PBS, (ii) polymer, and (iii) the complete structure (polymer+PBS), to gauge the effectiveness of the concept. Without either polymer layer or electrolyte, no signals were elicited. Current densities at short circuit (J_ph_) and photo-voltage at open circuit (V_ph_) were instead generated upon photoexcitation for the complete device (Fig. 2a,b). We illuminated the device for 300 ms with a high-speed white LED (5000 K) as falling within the ranges used for in vitro retinas and living cells light stimulation studies^12,14,27,32,45,46^ and below the limit allowed for prosthetic application^47,48^. The steady state photo-current after an initial spike is an evidence of a Faradaic process at the electrode/electrolyte interface^49^. Similarly to the concept of a DSSC^38^ charges are photogenerated in the semiconductor film, whilst the ions present in the biological liquid permit the exchange of charge at both the polymer/electrolyte and electrolyte/electrode interfaces enabling the presence of a sustained current. The platinum film catalyses charge transfer between the ions and the top transparent electrode. Current spikes visible when the system is perturbed^50,51^, i.e. when light is turned ON or OFF, are the result of a capacitive process due to electrostatic perturbation of accumulated carriers and ions with strong Coulomb interactions localized close to the polymer/electrolyte and electrolyte/metal interfaces (e.g. Helmholtz layers)^49^. Capacitive coupling is one of the most biologically safe mechanism used for cells and tissues electrical stimulation^15,52^. In this device the electrolyte maintains the physiological environment of biological cells and tissues (which would be present in an in vivo investigation or that could be potentially placed inside the device) and enables the movement and transport of charge carriers along the section of the device between photo-electrode and counter-electrode. J_ph_ values, referred to the steady state (at the half of the light pulse width, namely at 150 ms from the light switched ON) current signals are in the order of 0.17 ± 0.10 µA/cm^2^. J_ph_ spike values are in the order of 0.51 ± 0.28 µA/cm^2^ and of − 0.29 ± 0.12 µA/cm^2^ at the start and the end of the impulse of light respectively (Fig. 2a). Under the convention of positive J values typically used in physiology reporting, the negative V_ph_ signals decrease as a function of time upon light stimulation with V_ph_ values in the order of − 5.4 ± 1.8 mV at a radiant exposure of 53.4 J/m^2^ corresponding to 17.8 mW/cm^2^ for 300 ms (Fig. 2b). The elicited photo-responses were similar to those reported in patch-clamp electrophysiology^27^, contact pad^14^, and MEA^12^ experiments where the samples are immersed in an open electrolyte bath with a reference electrode. This demonstrates the successful operation of our bio-hybrid device architecture concept.

The opto-electrical response of the device was tested at different light power levels (Fig. 2c,d) ranging from 17.8 mW/cm^2^ (∼10 × 10^3^ lx) to 93.8 mW/cm^2^ (∼60 × 10^3^ lx) representing the intensity range typically experienced in human photopic vision outdoors^53^ (from 10 to 100 × 10^3^ lx) and the range of irradiance levels generally used for in vitro retinal stimulation experiments^12,14,47,54^. Currents (J_ph_ first peaks, Fig. 2c; J_ph_ second peaks Supplementary Fig. S4b; J_ph_ steady state Supplementary Fig. S4c) increase linearly with irradiance whereas V_ph_ max (measured after 300 ms from light switched ON) as function of the increasing irradiance levels shows a quasi-logarithmic trend (Fig. 2d). Considering possible future application of the bio-hybrid device for in vitro retinas stimulation, we also used shorter light pulse stimuli of 10 ms (optical pulse width for retinas stimulation^13,14^), to opto-electrically characterize the device (Supplementary Fig. S3). Recorded values of current capacitive peaks are similar with good approximation for 10 and 300 ms light pulse duration (Supplementary Fig. S4a,b). Instead, photo-voltage signals recorded as responses to 10 ms light pulse stimuli are of the order of 0.5 ± 0.2 mV and of 2.3 ± 0.6 mV for low (17.8 mW/cm^2^) and high (93.8 mW/cm^2^) irradiance levels, respectively (Supplementary Figs. S3b, S4d). These photo-generated low-voltage signals should still be sufficient for stimulation of retinas in vitro. In fact, as demonstrated in previous works, ~ 0.6 mV signals should be enough to elicit electrical responses in a blind chick retina^12^.

**Figure 3:**
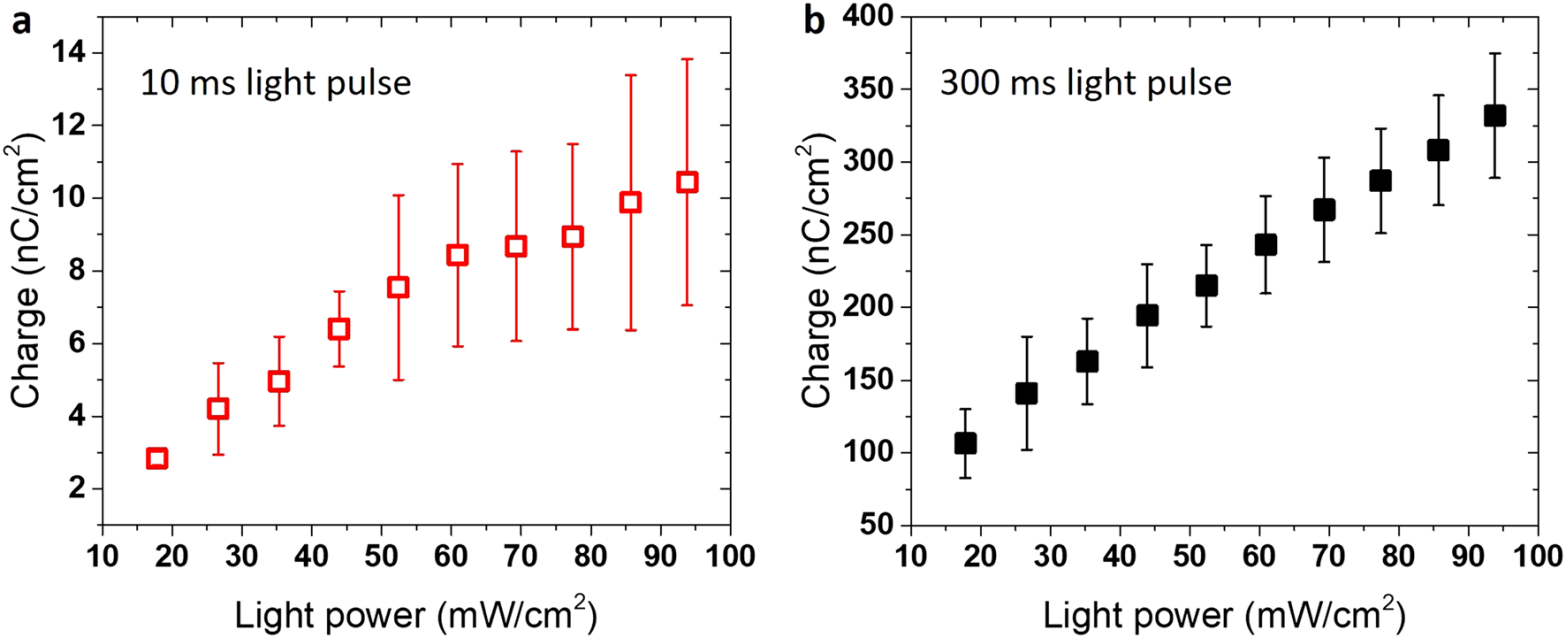
Total charge measured upon photo-stimulation. Photo-generated charge calculated from the time integral of the photocurrent response curves as a function of increasing light power for 10 ms (**a**) and 300 ms (**b**) light pulse duration. Samples n = 3. Y-error bar referred to the Standard Deviation (SD) are shown. (OriginPro 2016 was used).

We also examined the total charge generated by the device upon illumination. The total charge (calculated as the time integral of photocurrent) at different irradiance levels was 2.8 ± 0.2 nC/cm^2^ and 10.4 ± 3.3 nC/cm^2^ for 10 ms light pulse stimulation (Fig. 3a) and 106.4 ± 23.7 nC/cm^2^ and 331.9 ± 42.8 nC/cm^2^ for 300 ms light pulse stimulation (Fig. 3b) at 17.8 and 93.8 mW/cm^2^ respectively. Thus, the device is able to generate a charge level in the range of few nC/cm^2^ which is useful for neural stimulation and in particular for retinas stimulation (bipolar and ganglion cells)^55^. The total photo-generated charge consists of a capacitive and a faradaic component. While the capacitive charge corresponds to the current spikes generated during few ms after the light is switched ON and OFF and includes redistribution of charges at the polymer/electrolyte and electrolyte/metal interfaces, the faradaic component is related to the steady-state photocurrent and involves transfer of electrons across the electrode/electrolyte interface^51^. As suggested by previous works, faradaic reactions implicate reduction/oxidation reactions involving the oxidation of the organic semiconductor interfaced with the electrolyte solutions. Oxygen reduction and hydrogen peroxide production or hydrogen evolution reactions have been implied^56,57^. Transport of mobile ions present in the electrolyte solution across the device cross-section and charge-transfer at the electrodes are also processes that give rise to photo responses of this type^58^.

To gauge the lifetime of the device, we performed an accelerated ageing test. The sandwiched device containing PBS was subjected to continuous white light illumination at 17.8 mW/cm^2^ (density power typically experienced in human photopic vision outdoors^53^) and at close to 100 mW/cm^2^ (density power used to evaluate electrical performance in solar cells) and the opto-electrical responses of the device (at light stimuli of 300 ms, power levels ranging from 17.8 up to 93.8 mW/cm^2^) were monitored. Irradiance levels generally used for in vitro retinal stimulation experiments span from 1 to 94 mW/cm^2^^12–14^. For continuous light irradiation at 17.8 mW/cm^2^ photo-voltage electrical outputs did not change substantially (Supplementary Fig. S5a) up to 10 min, i.e. sufficient for testing the response of the devices, whilst the J_ph_ max decreased by 15% (Supplementary Fig. S5c). At the very high optical powers of 93.8 mW/cm^2^ degradation accelerated significantly: V_ph_ max diminished by 12% (Supplementary Fig. S5b) and J_ph_ max by 40% (Supplementary Fig. S5d) after 5 min. We continued the accelerated test at these high powers for 48 h noticing a reduction of the current by 64% from the fresh device and visible alteration of both the photo-electrode (formation of blisters and wrinkles) and counter-electrode (residues of the biological medium) as well as evaporation of the electrolyte (Supplementary Fig. S5e,f). This is not surprising as the test at the high irradiance of 93.8 mW/cm^2^ is the equivalent of looking straight at the sun which is known to damage the eye even after few seconds^59,60^. It would be interesting to carry out a long detailed study on lifetimes by varying systematically the irradiance levels and determine the threshold that leads to significant degradation on materials and electrolyte solutions in order to evaluate what changes are needed to improve the lifetimes of artificial retina systems.

### Pixelated polymer artificial retina via inkjet printing

**Figure 4:**
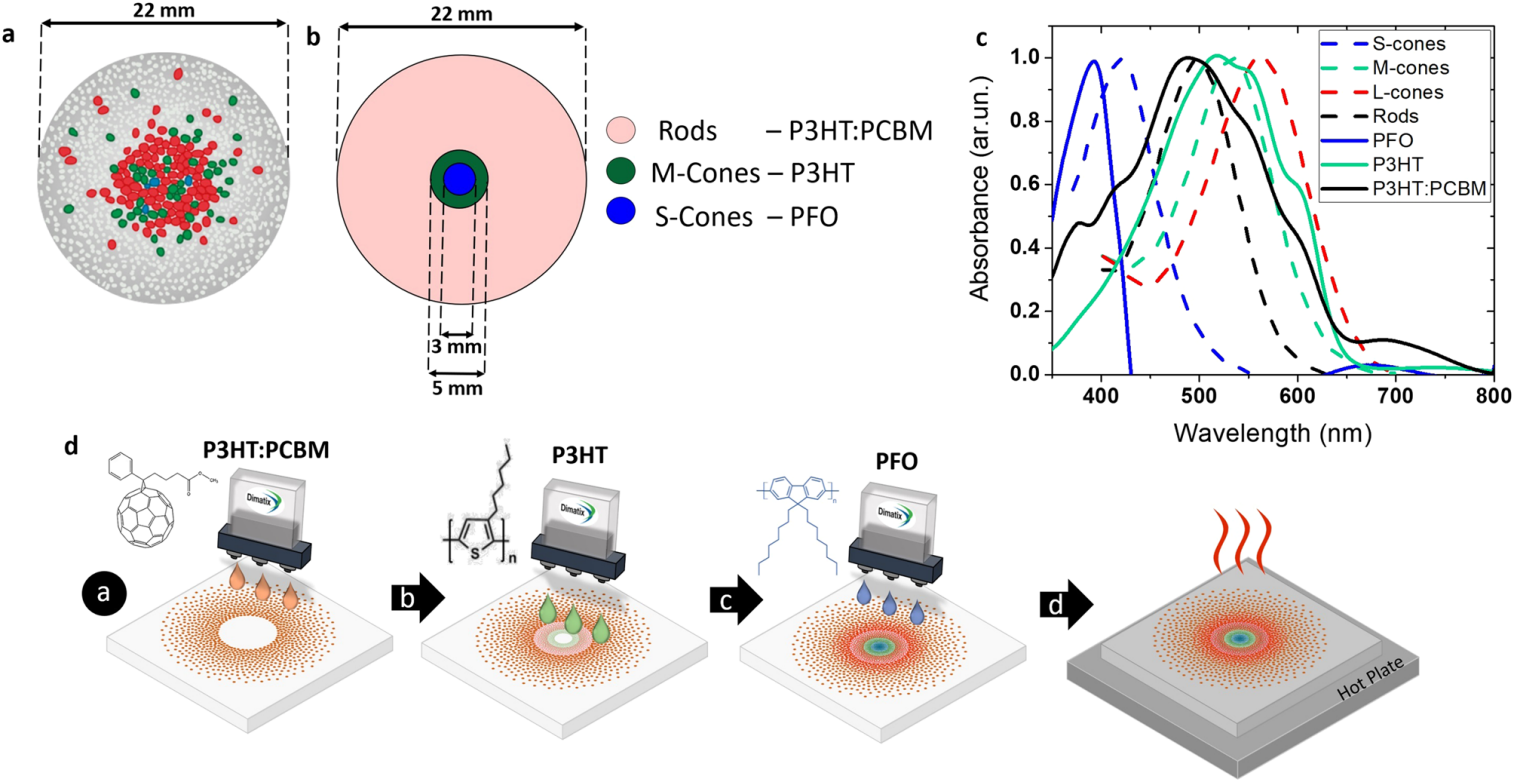
Model, materials and inkjet printing technique for the fabrication of the pixelated polymer artificial retina system. (**a**) Schematic representation of photoreceptors distributed on a human retina based on anatomical model. The S-Cones (blue), M-Cones (green) and L-Cones (red) are mostly packed into the fovea, while rods (grey) are located mostly in the periphery of the retina. (**b**) A proposed simplified concentrically organized artificial retina model to mimic the spectral response of the M-cones, L-cones and rods from mammalian retina. Concentric geometry model was used for ease of study. Rods-P3HT:PCBM annulus = 360.5 mm^2^; S-Cones annulus = 7.1 mm^2^; M-Cones annulus = 12.5 mm^2^. ((**a**,**b**) images created by using Microsoft PowerPoint). (**c**) Absorbance spectra of human photoreceptors (dashed lines: Rods, S-Cones, M-Cones, L-Cones) compared with those of polymers used in the pixelated inkjet-printed artificial retina of this work (continuous lines). Human photoreceptors absorbance from Bowmaker and Dartnall studies (OriginPro 2016 was used). (**d**) Schematics showing the inkjet printing process for the printed artificial retina. Chemical structures of conjugated polymers used for the artificial retina fabrication are shown: step (**a**), PCBM; step (**b**), P3HT; step (**c**), PFO. Step (**d**) represent the thermal annealing (Images created by using Microsoft PowerPoint).

Once the biocompatibility and the optoelectronic operation of the devices presented in Fig. 1 was demonstrated, the next step was to extend the single-polymer-layer device to a pixelated, colour sensitive, three-polymer-based device towards an artificial retina model. The size of a human adult retina is typically around 22.0 mm in diameter^61^. Photoreceptors are organized in a well-defined mosaic^62^ where rod densities are predominant in the peripheral retinal region while cones in the central part (fovea). Out of all cones, about 64% are L type, 32% M type and only 2% S type^63,64^. Relative ratio and position of cones varies greatly among different people even with regular vision^65^. Recently the concepts of pixelated visual implants have been exploited and retinal stimulating pixels (~ 100 µm in diameter) have been obtained by spin-coating, sputtering and photolithography techniques^14^. We designed a simplified model to fabricate the pixelated artificial retina system using the resulting anatomical map of the photoreceptors mosaic and placing locally different polymer semiconductors with specific absorbance spectra via printing techniques (Fig. 4a,b). Conjugated polymers have been previously proposed as promising materials for colour detection^30,31,33^ due to band gap and optical tunability. We firstly identified polymers with absorbance spectra similar to that of human photoreceptors (for human photoreceptors absorbance we referred to Bowmaker and Dartnall studies^66^) (Fig. 4c). Regio-regular P3HT polymer (see “Methods” section) and a blend of P3HT with [6,6]-Phenyl C61 butyric acid methyl ester (PC_61_BM) (P3HT:PCBM) were used, mimicking well the green cone and rods absorbance spectra, respectively. As a third photosensitive polymer we chose Poly(9,9-di-n-octylfluorenyl-2,7-diyl) (PFO), to mimic S-cones. Its peak is shifted significantly from both those of P3HT and the P3HT:PCBM blend and thus they are all spectrally well distinguishable. Then the biocompatibility was evaluated for the P3HT:PCBM and PFO layers (Supplementary Fig. S6a), as previously described for P3HT (Fig. 1). No significant differences in the proliferation rate expressed as cell doubling time (CTRL = 1.50 ± 0.02 day; P3HT:PCBM = 1.43 ± 0.04 day; PFO = 1.52 ± 0.14 day) and percentages of proliferating cells (T0: CTRL = 51.14 ± 2.06%; P3HT:PCBM = 49.91 ± 3.09%; PFO = 56.27 ± 2.35%; T3: CTRL = 47.58 ± 1.80%; P3HT:PCBM = 47.78 ± 1.70%; PFO = 46.95 ± 2.39%) (Supplementary Fig. S6b–e) were observed on the polymer layers compared to control. Cell death analysed at T0 and T3 was very low, with no differences between CTRL, P3HT:PCBM and PFO (Supplementary Fig. S6f,g). These results indicated no toxicity of all the polymer layers analysed. Thus, a pixelated retina system based on M-cones (P3HT), S-cones (PFO) and rods (P3HT:PCBM) was printed, providing a dichromatic inkjet-printed artificial retina model. The three-polymer bio-hybrid printed device mimics the dichromatic vision of most mammals including rodents often used in biomedical studies^67^ such as mice that only possess S- and M-cones^68^. Furthermore, it delineates a starting point for a future trichromatic human artificial retina system. Indeed, the device-concept can be easily extended to other polymers. For instance, a possible candidate to mimic human L-cones (maximal absorption at λ = 560 nm) could be Poly[N-9′-heptadecanyl-2,7-carbazole-alt-5,5-(4′,7′-di-2-thienyl-2′,1′,3′-benzothiadiazole)] (PCDTBT) which has an absorbance spectra maximum at 576 nm.

In this study we assembled a simplified model of the anatomical retinal scheme consisting in a concentric ring design where each annulus represents a different retinal photoreceptor region. This simplification made it possible to selectively shadow-mask the different annuli in order to test the spatial and spectral response of our pixelated device. The outermost annulus of 360.5 mm^2^ corresponds to rods (P3HT:PCBM), whilst the two central annuli correspond to the M-cones (P3HT) of 12.6 mm^2^ and the S-cones (PFO) of 7.1 mm^2^ areas respectively. Total diameter was 22.0 mm. Inkjet printing was used for selective deposition of the polymers (Fig. 4d) being a powerful technique that offers excellent design flexibility and precise control in material deposition^69^, enabling contactless, mask-free and digital patterning minimising materials usage and waste produced.

Semiconducting polymer inks formulation (molecular weight, concentration, solvents, viscosity and surface tension) and inkjet deposition represented an important part of creating a complete colour-sensitive artificial retina system. High viscosity, nozzle clogging, agglomeration, precipitation, and uncontrollable drying patterns are among the frequently challenges encountered with inkjet printing^70,71^. PFO, P3HT and P3HT:PCBM were dissolved in a mixture of chlorobenzene (CB) and tetrahydronaphthalene (THN) (1:1) to obtain inkjet-printable polymer inks. The combination of CB:THN—medium/high boiling point solvent mixture—serves two purposes. CB was used to achieve homogeneous film formation owing to excellent solubility of the chosen polymers in CB. THN (b.p. = 270 °C) was used to prevent the nozzles clogging and to provide reliable jetting over time. Inkjet parameters (pulse voltages, cartridge temperature, substrate temperature, drop spacing, waveform) were carefully adjusted in order to obtain stable droplet formation and to achieve rounded-pixels layout (see “Methods” section).

**Figure 5:**
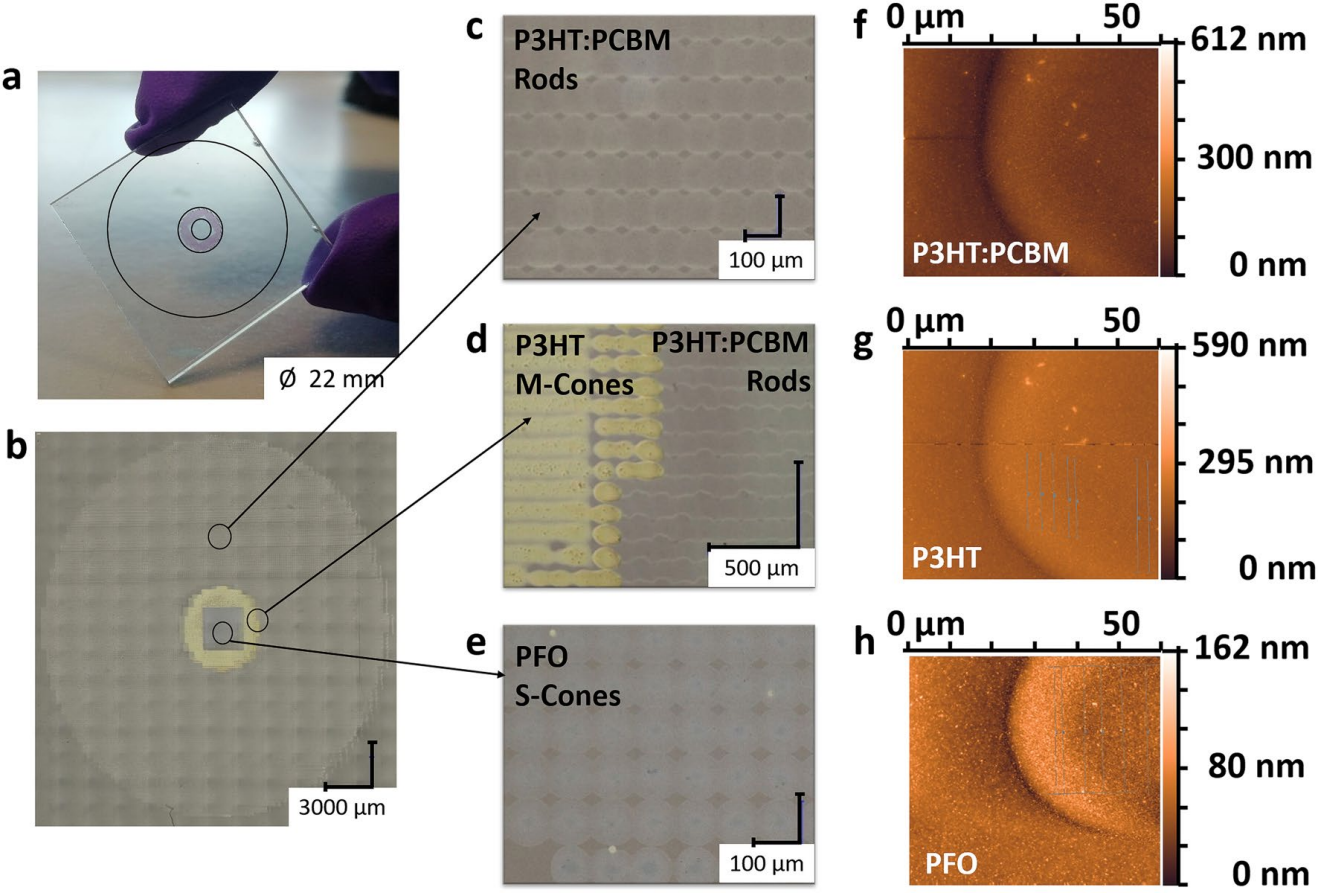
Inkjet-printed artificial retina device prototype and related optical microscopy analysis. (**a**) Device picture and (**b**) optical microscopy image of polymer inkjet-printed artificial retina showing, (**c**–**e**) different regions of the concentrically organized polymer pixels. (**f**–**h**) AFM images of photosensitive round polymer pixels. AFM characterisation was also used for thickness and roughness measurements of the pixels.

The artificial retina system was printed on Glass|FTO substrates. The inkjet-printed device consisted of ~ 42,100 pixels over the total area of 380.1 mm^2^. Optical microscope images show the concentric layout of arrays of small round polymer pixels (Fig. 5) with an average diameter size of 95 ± 5 μm and of 50 to 60 nm thickness as measured by Atomic Force Microscopy (AFM) with an average roughness of 6 to 8 nm (underlying Glass|FTO roughness was around 4 nm) and a packing density of ~ 110 pixels/mm^2^. The printed pixelated device is a starting point for higher pixel density artificial retina models. Indeed, to reduce the pixel size, it is possible to use additional layers to modify the wettability and surface energy of the substrate. An example is shown in Supplementary Fig. S7, where a spin coated P3HT:PCBM layer was used as a layer on which to print P3HT and PFO pixels that enabled us to obtain small diameters of ~ 50 μm. A different underlying layer with negligible optical absorption in the visible range would be suitable for this purpose. In general, inkjet printing equipment with smaller nozzle orifices/small droplet volume and higher viscosity inks along with substrate chemical surface functionalisation, can enable the realisation of pixel sizes smaller than 30 μm^72^. During printing from a single nozzle, individual sub-nanoliter ink droplets are ejected. The resulting drop diameter is related to the size of the nozzle orifice (1 pL drop can create a 20‐30 μm spot size) and final, dry, printed area size resulting from a single drop, can be adjusted significantly by the operating conditions^73^ and the surface treatment of the substrate. In order to approach the 2–10 μm size, typical for human photoreceptors^74,75^, alternative printing technologies can be adopted such as imprinting^76,77^, flexographic printing^78^, laser patterning^79^ or high resolution lithography techniques (optical and electronic).

To the best of our knowledge, no visual prosthetic devices can restore the natural colour vision to date. Our proposed concentric artificial retina model is simplified in order to enable demonstration of spatial resolution of the photo-response. Human cones are distributed radially from the fovea in a geometry that is unique from individual to individual. Even if it was not possible to achieve the ideal case of superimposing pixelated artificial photoreceptors with the same dimension and in the same position as that of the real retina it wants to mimic (e.g. for implantation), there remains possibilities for inducing and possibly restoring some trichromatic vision to the blind. For example, it would be convenient to identify local density variations of cone types across a region of the retina and to place the larger pixels of one type of polymer over the areas where there are larger concentrations of the homologous cones. The avenues for exploration and increasing understanding of the visual system are many.

**Figure 6:**
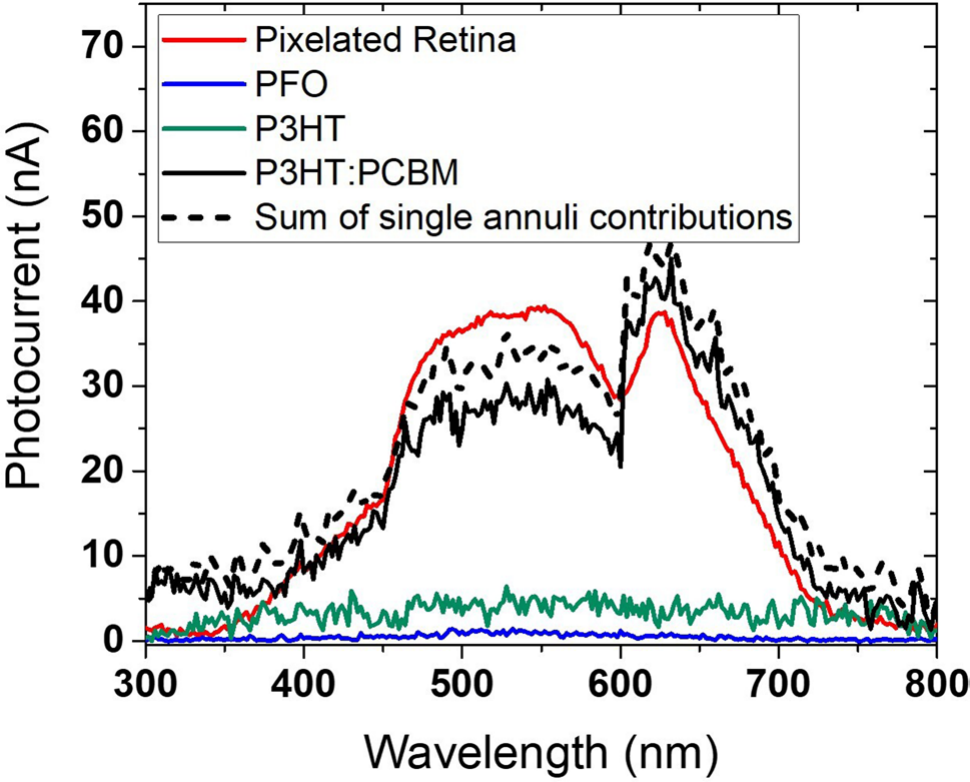
Spectral responsivity of the inkjet-printed artificial retina. Photocurrent measured from the pixelated retina compared to the photocurrent calculated as the weighted linear summation of the single polymer-annuli contributions. I_ph_ originating from singular annuli were recorded by using a monochromator/lock-in set up and additional customized shadow masks so as to illuminate only the polymer annulus of interest by covering the remaining part of the printed artificial retina. (OriginPro 2016 was used).

The artificial retina device was completed by assembling a top transparent platinized counter-electrode with a 60 µm thick thermoplastic spacer and filling the space with PBS. It was then characterised by placing it in a customized black box and illuminating the whole active area (380.1 mm^2^) via a train of 6 light pulses using a LED lamp, 6 mW/cm^2^ at 5 cm distance (standard warm white light spectrum, Supplementary Fig. S8). Chronoamperometric J_ph_ curves (impulses of 2 s light ON, 2 s light OFF) are consistent with those measured for the prototypical single layer device (Fig. 2a,b), showing a capacitive switch and a more steady contribution to the current with J_ph_ ranging from 0.2 to 0.3 μA/cm^2^ (Supplementary Fig. S9a). On average responses were 7% greater when light was shone from the bottom of the polymer layer rather than through top platinized electrode, likely due to the absorption of the electrolyte and counter-electrode (Supplementary Fig. S9b). Photovoltage data were recorded with a train of white light pulses of 5 s duration followed by 60 s dark period. A significantly high V_ph_ of 12.0 mV was recorded after a 5 s long light stimulus (Supplementary Fig. S9c) (it was ∼0.3 mV and ∼1.1 mV after 10 ms and 300 ms respectively, thus consistent with short light pulse stimuli).The darkness period was extended to 60 s to enable V_ph_ to recover within 10% of its initial value. It would be interesting to evaluate the optoelectrical responses of a single polymer pixel in order to assess whether the one-polymer pixel/one-photoreceptor stimulation is possible. A low-level electrical measurement set-up with a current resolution of less than 1 pA would be required for this analysis. Thus, this will be part of a future investigation. With our current systems we were able to gauge the spectral and spatial responsivity of the different polymer regions of the pixelated device by masking/unmasking larger annuli regions (see “Methods” section). The resulting photocurrent (I_ph_) as a function of wavelength of the light source is shown in Fig. 6 with the same measurements carried out on each of the single polymer (or blend) annulus of the printed artificial retina. Customized shadow masks were used to exclusively illuminate the polymer annulus of interest. Notwithstanding the geometrical domination of the P3HT:PCBM component (PFO 2.0%, P3HT 3.3%, P3HT:PCBM 94.7% of total inkjet-printed artificial retina area), each pixelated polymer annulus contributes to the overall I_ph_ signal (Fig. 6, Supplementary Fig. S10). The pronounced red peak in the P3HT:PCBM spectrum can be due to various effects including layer thickness^80^, solvent used^40^, thermal treatment^81^ or the interaction with materials such as dopants^82^ as well with components of the electrolytic medium.

## Discussion

The main focus of this study was the development of a conjugated polymer-based bio-hybrid artificial retina model to study photo-response properties of different band-gap polymer pixels, interfaced with electrolyte medium, aiming to mimic the colour response of mammalian cones and rods, from the single-polymer-layer device concept to a fully pixelated system. The device layout consists of two transparent, planar and parallel electrodes: the P3HT-spin-coated photosensitive working electrode and the Pt-screen printed counter-electrode. Planar polymer layer for retinal opto-electrical stimulation have been already successfully demonstrated in literature, both as polymer thin films spin-coated on conductive glass substrates and as polymer-coated MEAs electrodes^12–15^. In these works patch-clamp^27^, contact pad^14^, and MEA^12^ electrodes are interfaced with electrolyte in an open container configuration with a metal counter-electrode immersed in the electrolyte. Distinctively, the presented bio-hybrid device enables the study of the spectral and spatial responses of the substrate/polymer system interfaced with biological electrolyte within a closed architecture. Consistently with other works on conjugated polymers^12,13,15,29,32,33,46^, the fabricated bio-hybrid photo-electrode shows good biocompatibility. Thus, the device can represent a useful tool for studying light stimulation and recording of bio-electrical signals. The fabricated bio-hybrid device response to incident light elicits transient photo-voltage (mV) and photo-current (µA/cm^2^) outputs that resemble those seen in patch-clamp and MEA experiments and that would suffice to elicit a response in a retina^83^ or in neurons^84^. Even if capacitive coupling (0.51 ± 0.28 µA/cm^2^ and of -0.29 ± 0.12 µA/cm^2^ spikes at the beginning and the end of light stimuli, 17.8 mW/cm^2^) is the dominant process in the device upon illumination, a minor faradaic component (0.17 ± 0.10 µA/cm^2^) is present. Clearly charge balancing is a vital process to ensure the electrode–tissue interface safety operation. However, we think that self-regulation of ionic gradients in living systems could help to prevent tissues and cells damage over time^85^. Thus, we can accept the recorded current density signals and faradaic component values with a good margin of tolerance also in respect to similar work found in literature where faradaic processes were not completely excluded^13–15^. The bio-hybrid device presents some advantages compared to electrophysiological investigative systems: easy-to-handle and transportable closed system, controllable size, and a required small amount of electrolyte. It permits the utilization of tools from an electronic engineering/physics/chemistry laboratory rather than the more sophisticated tools found in bio-medical laboratories. In addition, the device architecture opens the possibility of inserting biological materials (cells or tissues) in the chamber of a compact device filled with the biological electrolytic medium. Limitations of this device compared to the open immersions used in typical MEA or electrophysiology experiments, may be represented by the constraints in inserting fairly large tissues, such as retinas, in the small chamber for their study, as well as the large dimensions of the current electrodes used (see Fig. 1a,b). The latter may need to be patterned in a smaller size (i.e. similar to those found in MEAs, i.e. of the order of ≤ 0.1 mm) and be more numerous for better efficiency to record bioelectrical signals from local areas of live retinas. It will also be critical to devise ways to keep living samples functioning in a restricted environment which may require the introduction of inlet and outlet orifices for perfusion. Until these evolutions are implemented and tested, the device can be useful to spatially and spectrally investigate photo-responses of semiconductor films or patterns immersed in physiological fluids that mimic those found in living organisms.

The device was also fabricated via inkjet printing technique. Thus, an inkjet-printed colour-sensitive artificial retina device consisting of three different types of semiconducting round polymer pixels, with distinct absorption spectra, mimicking the chromatic sensitivity of photoreceptors in the eye and interfaced with a physiological medium was demonstrated. The number of pixels was 42,100, the density of the artificial photoreceptors was ∼11,000 pixels/cm^2^ and the corresponding spatial resolution was 267 dpi (dots per inch), with pixel diameters of 95 ± 5 μm. Printing technologies enable placement of different materials in the locations of choice. In the future, with higher resolution techniques being developed (i.e. ≤ 10 µm), one could first scan/image an individual retina and then print the pixels spatially where exactly one wants to place them and this can be done for each individual retina (more costly if one uses photolithographic techniques). Secondly, printing techniques enable deposition on uneven or curved surfaces which can be beneficial for artificial retina concepts. The polymer pixelation allows a light-sensing device to spectrally and spatially resolve incoming light providing photo-responses that are both chromatically and spatially sensitive. The optoelectrical characterization of a single polymer pixel awaits further investigation. Future studies can be devoted in replacing glass with flexible substrates such as PET, poly (dimethylsiloxane) (PDMS), and silk fibroin films, the latter of which are implantable in vivo^46^. Moreover, additional studies on the device biocompatibility and its possible integration with healthy or degenerated explanted retinas from animal models are necessary to demonstrate future optoelectronic applications in the field of retinal prostheses.

## Methods

### Substrates preparation for bio-hybrid interface

Transparent Glass|FTO substrates (2.2 mm thickness) were cut in 2.5 cm by 2.5 cm and cleaned by subsequent rinses in ultrasonic bath using deionized water, pure acetone, isopropyl alcohol (5 min each). Then dried with an air gun.

### Polymer formulations for bio-hybrid interface

rr-P3HT (Rieke Metals, Inc.) (regio-regularity ≥ 95%, molecular weight: 51,000 g/mol) was used without any further purification. Polymer solutions in chlorobenzene (30 mg/mL) were prepared by heating polymer and solvent mixture at 100 °C for 5 min and then stirring at 50 °C overnight. rr-P3HT-chlorobenzene solution was spin-coated on FTO glass substrates (2000 r.p.m., rotation duration 60 s) inside the glovebox (Mbraun Inc.). Organic thin films were annealed on a hotplate (120 °C, 2 h). To obtain a square active area of 0.8 cm by 0.8 cm, the excess of polymer was removed using p-Xylene solvent and a polymer protective film mask.

### Electrolyte compartment for bio-hybrid interface

Thermo-plastic sealant (Surlyn, 60 µm thickness, DU PONT) masks, creating the milli-volume chamber, were designed to cover the frame of the 0.8 cm by 0.8 cm active area on the device and cut using a cutting machine (Small-one, magicut cutting plotter). Two orifices placed in opposite sides were designed to fill electrolyte inside the device. Masks were pre-attached on the substrates via pneumatic heat press (model Special) (90 °C, 0.5 bar pressure).

### Counter-electrode for bio-hybrid interface

A transparent platinum layer was screen-printed on Glass|FTO substrates (2.5 cm by 2.5 cm). After platinum paste precursor (3D-nano) deposition, the wet platinum layers were processed by a firing process (30 min, 480 °C).

### Electrolyte solution

Phosphate buffered saline solution (Gibco) was used as electrolytic solution. PBS is a balanced salt solution containing: CaCl_2_ (0.9 mM), MgCl_2_ (0.5 mM), KCl (2.6 mM), KH_2_PO_4_ (1.5 mM), NaCl (137.9 mM) and Na_2_HPO_4_ (8.0 mM) dissolved in double distilled H_2_O. PBS was filled inside the device using a 1 mL syringe by the orifices present in the Surlyn masks. Devices were then closed using a biphasic glue.

### Inkjet-printed artificial retina fabrication

FTO glass substrates (2.5 cm by 2.5 cm) were treated with a standard cleaning process as described before. They were then treated with Argon/Oxygen plasma (100 W, 10 min) and UV treatment (1000 W, 5 min) to improve wettability and hydrophilicity. A model design reproducing the photoreceptors distribution on the human retina was designed and drawn via a standard vector (Inkscape) and raster (GIMP) graphics software. Polymer inks were formulated for each annulus of the retina devices and printed. Chlorobenzene:Tetrahydronaphthalene (1:1) mixture was used as solvent for polymer inks formulation. P3HT:PCBM, PFO, P3HT were diluted to a concentration of 6% w/w, 3% w/w, 6% w/w respectively. Inks were prepared by heating a polymer and solvent mixture at 100 °C for 5 min and then stirring at 50 °C over night. Each polymer ink viscosity was experimentally determined using a viscometer and found to be 4.5 mPa s (20 °C), 3.7 mPa s (20 °C) and 4.7 (20 °C), respectively. A Dimatix Materials Printer DMP-2830 (FUJIFILM Dimatix Inc.) was used for the printing process. rr-P3HT (regio-regularity higher than 95%, m.w.: 51,000 g/mol) was purchased from Sigma Aldrich; PC61BM (m.w. 910.88 g/mol, purity ≥ 99.5%) from Sigma Aldrich; and PFO (m.w. 40–150,000 g/mol) from American Dye Source, Inc. All the inks were sonicated at 37 °C, 100% power for 40 min. Then inserted in the print cartridge (Dimatix DMC-11610, 10 pL piezo driven jetting cartridge) using a 0.22 µm pore size hydrophilic polyether sulfone filter. Cartridges were fixed to a print head consisting of 16 nozzles (21 μm in diameter). Single nozzle jetting was used. Cartridge temperature was fixed to 30 °C. Firing Voltage was set to 20 V, 40 V and 26 V for P3HT:PCBM, PFO and P3HT polymer inks respectively. Maximum jetting frequency was established to 2.0 kHz for P3HT:PCBM and P3HT inks and to 3.0 kHz for PFO ink. Inkjet-printed artificial retina samples were finally annealed (2 h, 120 °C). For polymer absorbance spectra measurements, polymer inks were spin coated (2000 r.p.m., rotation duration 60 s) on previously cleaned Glass|FTO substrates.

### Counter-electrode for the inkjet-printed artificial retina

A platinum layer was deposited on Glass|FTO substrates (2.5 cm by 2.5 cm) via blade coating and fired at 480 °C for 30 min. Thickness of the platinum layer was less than 1 µm, as the same obtained via screen printing technique.

### Morphological characterization

The thickness of polymer thin films and polymer-pixels were measured through a profilometer (Bruker Dektak XT Stylus Profilometer). Atomic Force Microscope (AFM—NT-MDT Solver NEXT equipped with an S7 scanner with a scan area of 5 µm by 5 µm) was used for the morphology investigation of polymer rounded pixels. A minimum of three separate scans were recorded for each sample. The AFM data were analysed with SPM software Gwyddion v2.51. Optical microscope (Leica DM 2500 optical microscope) was used for the morphological characterization of the pixelated artificial retina device. Confocal microscope (Olympus Lext—3100) was used for aged samples morphology analysis. All measurements were performed at ambient conditions.

### Cell culture and cell adhesion

Human Neuroblastoma SH-SY5H cells (ATCC) at 2 × 10^4^ cell/cm^2^ density were plated on the active area of the device (Glass|FTO|P3HT, Glass|FTO|P3HT:PCBM, Glass|FTO|PFO) (P3HT, P3HT:PCBM, PFO) placed in a polystyrene tissue-culture treated dish or plated directly on a polystyrene tissue culture treated dish (Easy Grip, Falcon) as control (CTRL). Cultures were carried out in DMEM (Gibco) supplemented with 15% Fetal Bovine Serum (FBS, Gibco), 2 mM l-glutamine, 100 UI/ml penicillin-G and 0.1 mg/ml streptomycin (all from Sigma-Aldrich) at 37 °C, 5% CO_2_ in 95% humidified incubator. To promote cell adhesion, polystyrene dish and polymer surface were coated with 0.7% w/v gelatin (from porcine skin, Sigma Aldrich) in ddH_2_O for 10 min at 37 °C. About 16 h (overnight) after cell seeding, the culture medium was replaced and the adhered cells where either analyzed immediately (T0) or cultured for additional 3 days (T3), depending on the experimental setting. For the adhesion analysis, cells were detached by Trypsin–EDTA 1× solution (Sigma-Aldrich) at T0 and counted in a Bürker chamber.

### Cell proliferation and cell viability

For the analyses of cell proliferation and viability, the numbers of cells seeded on P3HT, P3HT:PCBM and PFO layers were adjusted based on their rate of adhesion compared to control (standard plastic plate) in order to have at T0 the same number of adhered cell in all experimental conditions (Normalized adhesion: CTRL = 1.49 ± 0.38 × 10^4^ cell/cm^2^; P3HT = 1.40 ± 0.29 × 10^4^ cells/cm^2^; P3HT:PCBM = 1.45 ± 1.00 × 10^4^ cell/cm^2^; PFO = 1.23 ± 0.89 × 10^4^ cell/cm^2^). For proliferation analysis, cells cultured on standard plastic plate, on P3HT, P3HT:PCBM or PFO device were detached by Trypsin–EDTA 1× solution (Sigma-Aldrich) at 0, 1 and 3 days of culture and counted in a Bürker chamber. GraphPad Prism (software version 7.0, San Diego, CA) was used to calculate the doubling time, i.e. the time needed to double the cell number. The percentage of proliferating cells was estimated using the Click-iT Plus EdU Cell Proliferation Imaging Kit, Alexa Fluor 488 dye (Molecular Probe, Cat. n. C10637) according to the manufacturer’s instructions. Briefly, cells were incubated with the EdU solution (10 μM) for 2 h at 37 °C and 5% CO_2_ in 95% humidified incubator. Cells were then fixed with 4% formaldehyde for 15 min, permeabilized with 0.5% Triton X-100 in PBS for 20 min and stained with Click-iT Plus reaction cocktail for 30 min at room temperature. After washing three times with PBS, cells were observed by Leica DMI6000B microscope combined with a digital camera, and fluorescent and phase contrast micrographs were merged in order to perform the analysis. The cells were counted with the Image J software and the percentages of EdU positive cells (green) compared to total cells (EdU positive cells + EdU negative cells) in at least three randomly selected fields were calculated. Cell viability was analyzed by staining cells with ReadyProbes Cell Viability Imaging Kit following manufacturer instruction. Briefly, cells nuclei of dead cells were stained with NucGreen Dead reagent. Leica DMI6000B microscope combined with a digital camera imaged the cells, and fluorescent and phase contrast micrographs were merged in order to perform the analysis. The number of death and total cells were counted with the Image J software in at least three randomly selected fields and the dead cells expressed as percentage of total cells.

### Statistical analysis

Data were analyzed with GraphPad Prism (software version 7.0, San Diego, CA). Results were given as means ± SEM of at least three experiments and P value was determined by one-way Anova and Bonferroni post-analyses. The level significance was set at P < 0.05 (*, a), P < 0.01 (**, b), P < 0.001 (***, c) and P < 0.0001 (****, d).

### Measurement setup

Three measurements set ups were used. ARKEO apparatus (Cicci Research s.r.l.) was used to opto-electrically characterize the bio-hybrid interface. Transient photovoltage (TPV) and charge extraction (CE) software routines were used. Light stimuli (from the bottom of the device) of 10 and 300 ms duration after 100 ms of dark were presented every 60 s with increasing light intensities (17.8, 26.6, 35.3, 43.9, 52.4, 60.9, 69.3, 77.4, 85.7, and 93.8 mW/cm^2^). Each measurement was done immediately after the electrolyte injection in the device. Plotted signals are the mean over 3 samples. Each measurement represents an average of twenty consecutive sweeps (10 kHz and 1 kHz sampling rate for 10 ms and 300 ms light stimuli pulse duration). In TPV recordings a baseline potential was removed to evaluate the potential difference (from dark to light). A 6 W LED lamp (6 mW/cm^2^, at 5 cm distance from the lamp, as measured with ThorLabs PM100D broadband power meter) driven by a microcontroller (schematics and connection in Supplementary Fig. S11) was used for pixelated device characterization. A train of 6 light pulses (alternating periods of 2 s light ON and 2 s OFF for J_ph_ measurements, and 5 s ON and 60 s OFF for V_ph_ measurements) was used. A Keysight precision current–voltage analyser (Keysight technologies) was used to measure the J_ph_ and V_ph_ signals from the inkjet-printed artificial retina device (5 Hz sampling rate). I_ph_ and V_ph_ across different wavelengths were recorded by using a light source filtered through a monochromator before been focused on the polymer film/inkjet-printed artificial retina device. Data were collected at 2 nm intervals, with scans conducted from 300 to 800 nm. Monochromatic light was narrow-band, with approximately 3 nm FWHM. The light source was chopped by a mechanical chopper at 70 Hz, whose reference signal was fed to a lock-in amplifier (0 db gain). All measurements were taken at room temperature. To examine if the electrical signals drift over time, a device-tester with a P3HT thin film and PBS was stimulated via a 530 nm wavelength light chopped at 70 Hz for ca. 2 min. Monochromator/lock-in set up was used to record electrical signals. No evident dependence on time was observed.

J_ph_, I_ph_ and V_ph_ generated were measured between platinum electrode (counter-electrode) and polymer electrode (photo-electrode) in short circuit (V_applied_ = 0 mV) and open circuit (I_applied_ = 0 mA) respectively. The photo-electrode was grounded.

The power and spectra of ARKEO fast speed LED (5000 K) were measured using a spectrometer (AVASPEC) and an integrating sphere (AVASPHERE-50-IRRAD). The 6 W LED lamp spectra were measured using an Ocean Optics Optics HR2000 + high resolution spectrometer (Ocean Optics, Inc.).

Data were analysed with OriginPro 2016.

## Supplementary information

Supplementary Information.

## Data Availability

Authors declare that all relevant data supporting the findings of this study are available in this paper and in its Supplementary Information file. Access to our raw data can be obtained from the corresponding authors upon reasonable request.
