# Supplementary material for: Colour-sensitive conjugated polymer inkjet-printed pixelated artificial retina model studied via a bio-hybrid photovoltaic device
Source: Sci Rep. 2020 Dec 8;10:21457. doi: 10.1038/s41598-020-77819-z (PMC7722856; doi:10.1038/s41598-020-77819-z)
Supplement: Supplementary file 1 — Supplementary Information. [file 41598_2020_77819_MOESM1_ESM.docx]

**Colour-Sensitive Conjugated Polymer Inkjet-Printed Pixelated Artificial Retina Model studied via a Bio-hybrid photovoltaic device.**

Manuela Ciocca^1,2^, Pavlos Giannakou^2^, Paolo Mariani^1^, Lucio Cinà^3^, Aldo Di Carlo^4,1^, Mehmet O. Tas^2^, Hiroki Asari^5^, Serena Marcozzi^6^, Antonella Camaioni^6^*, Maxim Shkunov^2^*, Thomas M. Brown^1^*.

^1^ Department of Electronic Engineering, University of Rome Tor Vergata, Via del Politecnico 1, Rome, Italy

^2^ Advanced Technology Institute, Department of Electrical and Electronic Engineering, Faculty of Engineering and Physical Sciences, University of Surrey, Guildford, UK

^3^ Cicci Research srl. Via Giordania 227, Grosseto, Italy

^4^ Istituto di Struttura della Materia, CNR-ISM, via Fosso del Cavaliere 100, Roma, Italy

^5^ European Molecular Biology Laboratory, Epigenetics and Neurobiology Unit, Via Ramarini 32, Monterotondo, Italy

^6^Department of Biomedicine and Prevention, University of Rome Tor Vergata, Via Montpellier 1, Rome, Italy

Correspondence and requests for materials should be addressed to T.M.B. (email: thomas.brown@uniroma2.it), M. S. (m.shkunov@surrey.ac.uk) and A.C. (camaioni@uniroma2.it).


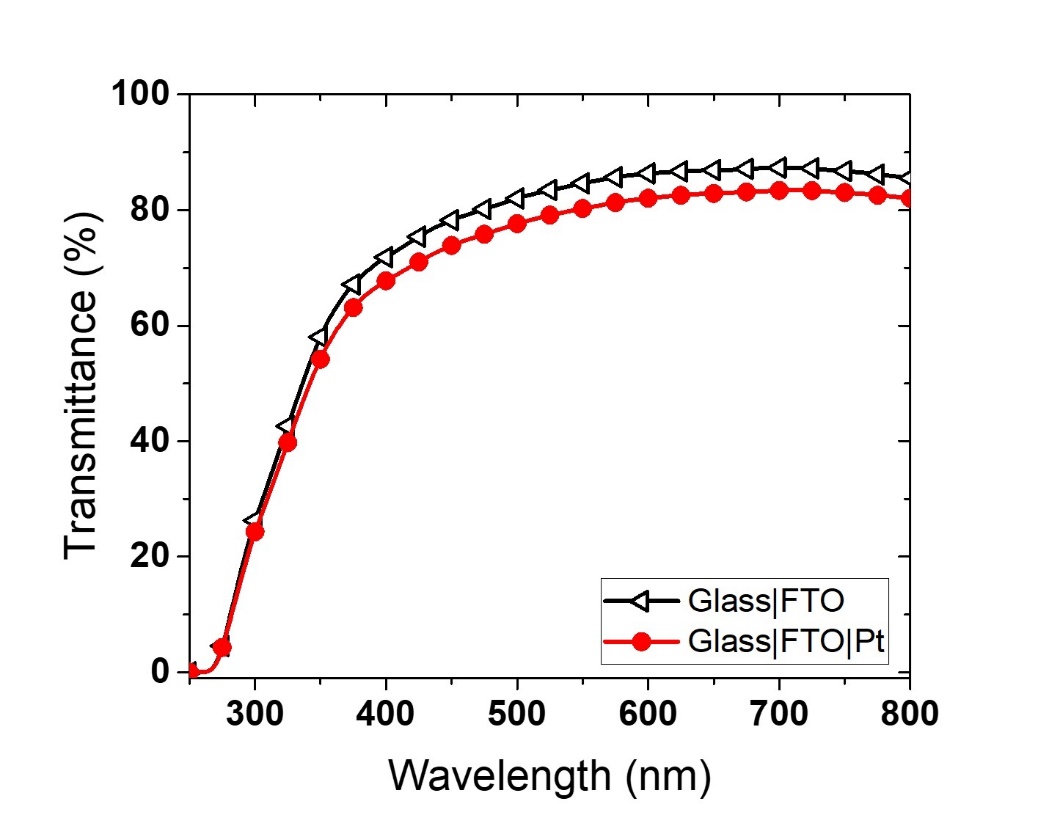


**Supplementary Fig. 1****:** **Transmittance of Platinum layer screen–printed onto Glass|FTO (red line) compared with bare Glass|FTO (black line). The average transmittance between 400 and 700 nm is 83% and 79% for Glass|FTO and Glass|FTO|Pt respectively. At 555 nm transmittance results 85% and 80% for Glass|FTO and Glass|FTO|Pt respectively. (OriginPro 2016 was used)**


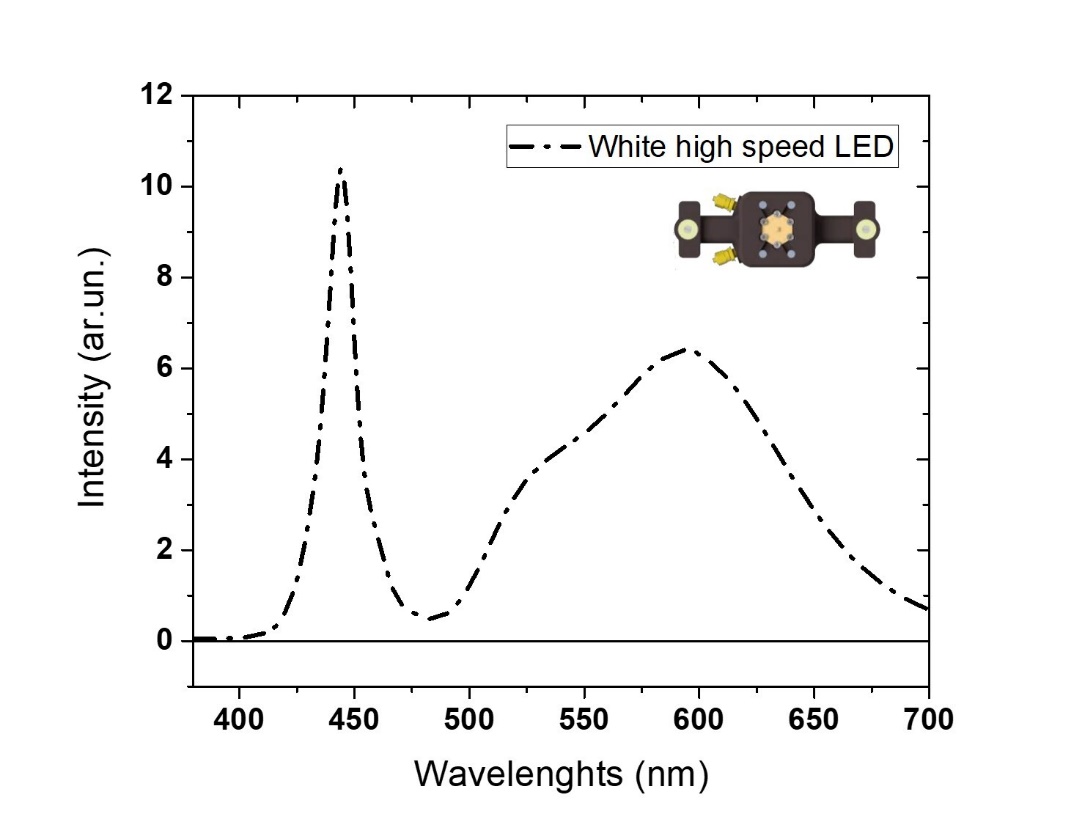


**Supplementary Fig. 2: White high-speed LED spectrum. LED used for light stimulation embedded in ARKEO system. (OriginPro 2016 was used).**


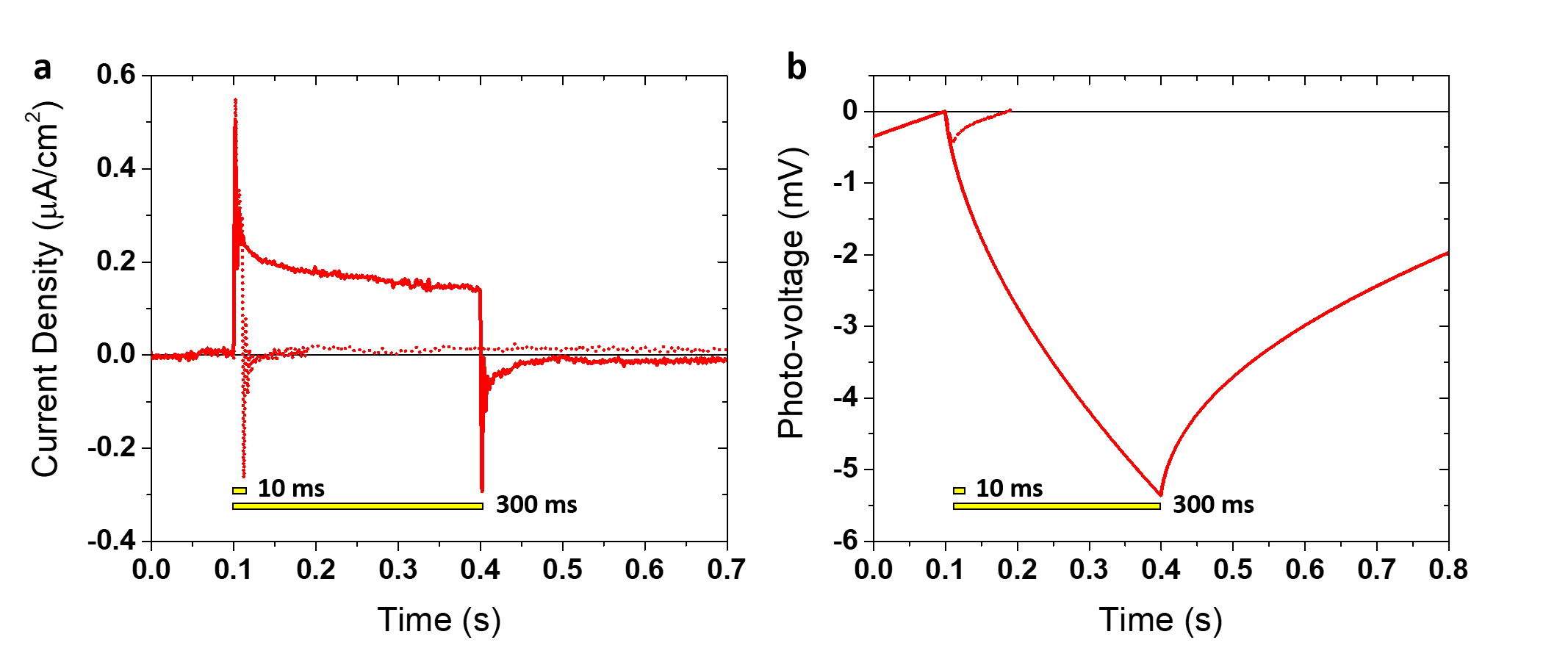


**Supplementary Fig. 3:** **Bio-hybrid device optoelectrical characterization. a)** **Transient current density generated by 10 ms and 300 ms white light stimulus (17.8 mW/cm^2^). b) Transient Photo-voltage recorded from the device under 10 ms and 300 ms white light (17.8 mW/cm^2^) stimulus. (OriginPro 2016 was used)**


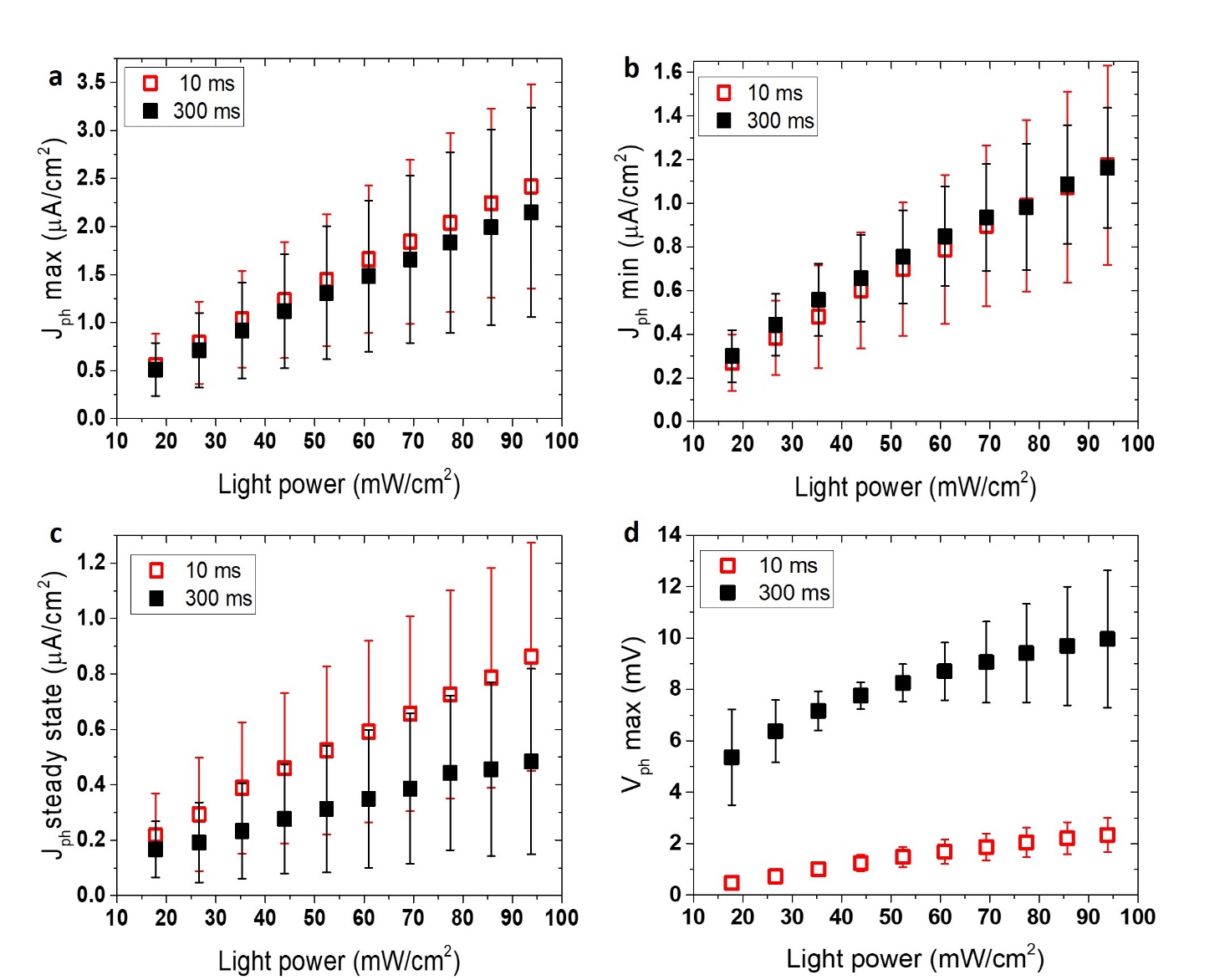


**Supplementary Fig. 4: Current density and photo-voltage dependence with irradiance levels, for 10 ms and 300 ms light pulse duration. Current density values as dependence on light power for a) first positive peak, b) second negative peak (absolute value) and c) steady state current signals. d) Photo-voltage as function of increasing light intensities. V_ph_ values (in absolute value) are referred to the value reached after 10 ms and 300 ms from the light switched ON. Mean values on samples n=3 are shown with Y-error bar referred to the Standard Deviation (SD). Radiant intensities used for J_ph_ and V_ph_: 17.8, 26.6, 35.3, 43.9, 52.4, 60.9, 69.3, 77.4, 85.7, and 93.8 mW/cm^2^ (high-speed withe LED -ARKEO set-up).** **(OriginPro 2016 was used)**


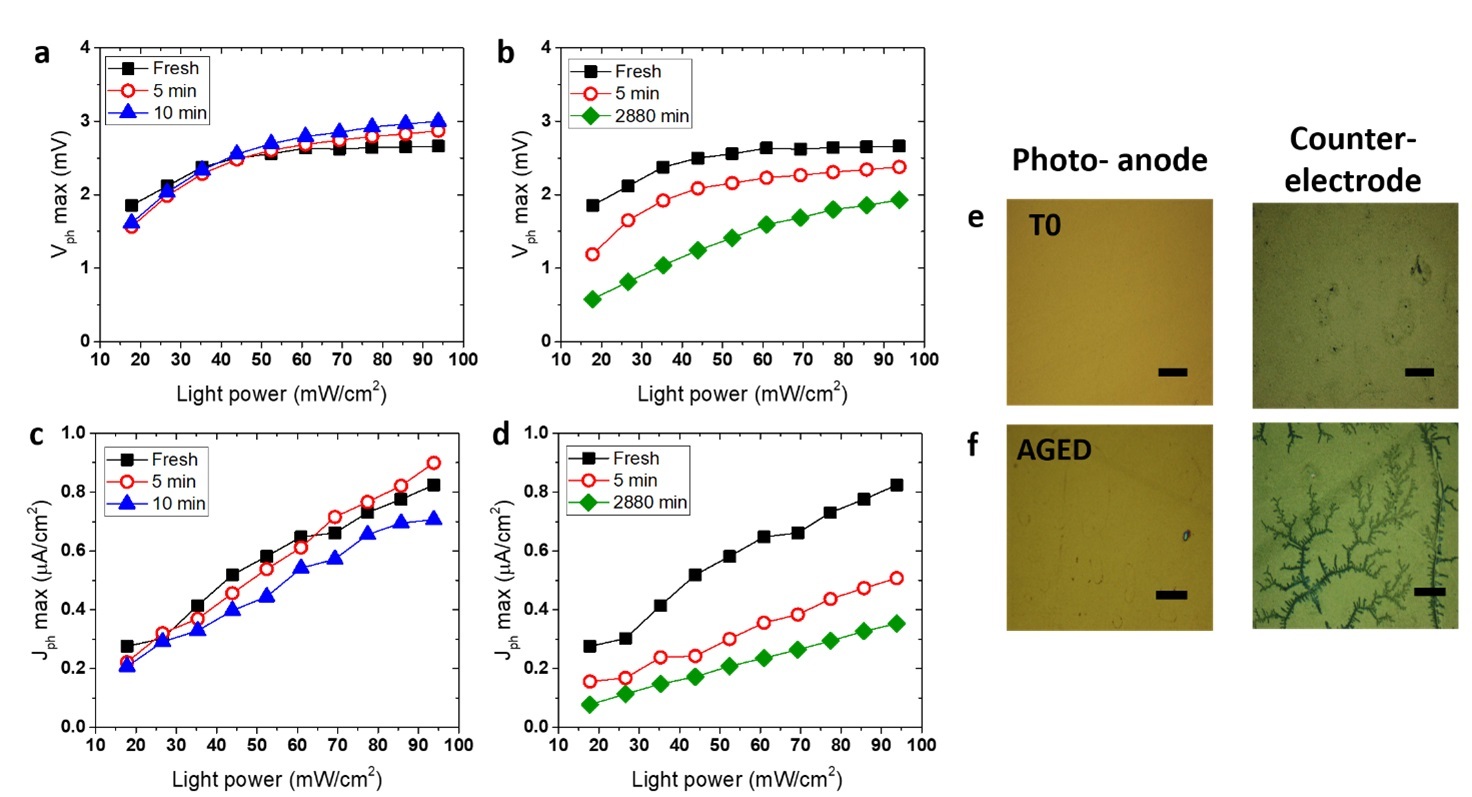


**Supplementary Fig. 5: Bio-hybrid device ageing tests. a) V_ph_ max (absolute value) reached at 300 ms from the light ON (namely when the LIGHT is turned OFF) monitored for freshly fabricated device and up to 10 minutes of continuous light stimulation (17.8 mW/cm^2^). b) V_ph_ max (absolute value) reached at 300 ms from the light ON (namely when the LIGHT is turned OFF) monitored for freshly fabricated device and up to 2880 minutes of continuous light stimulation (100.0 mW/cm^2^). c) J_ph_ max (first capacitive peak) reached when the light is switched ON, monitored for freshly fabricated device and up to 10 minutes of constant irradiation at 17.8 mW/cm^2^ and d) up to 2880 minutes of constant irradiation at 100.0 mW/cm^2^. (OriginPro 2016 was used for a, b, c, d images). e) P3HT- thin film device photoanode and Pt device counter electrode morphology at T0 (fleshly fabricated) and f) after 48 h under continuous illumination (100.0 mW/cm^2^) (AGED).** **Aged photo-anode presents formation of blisters and wrinkles; Pt-layer presents crystallized prints due to PBS evaporation. Confocal microscope was used. Scale bar 50 um.**


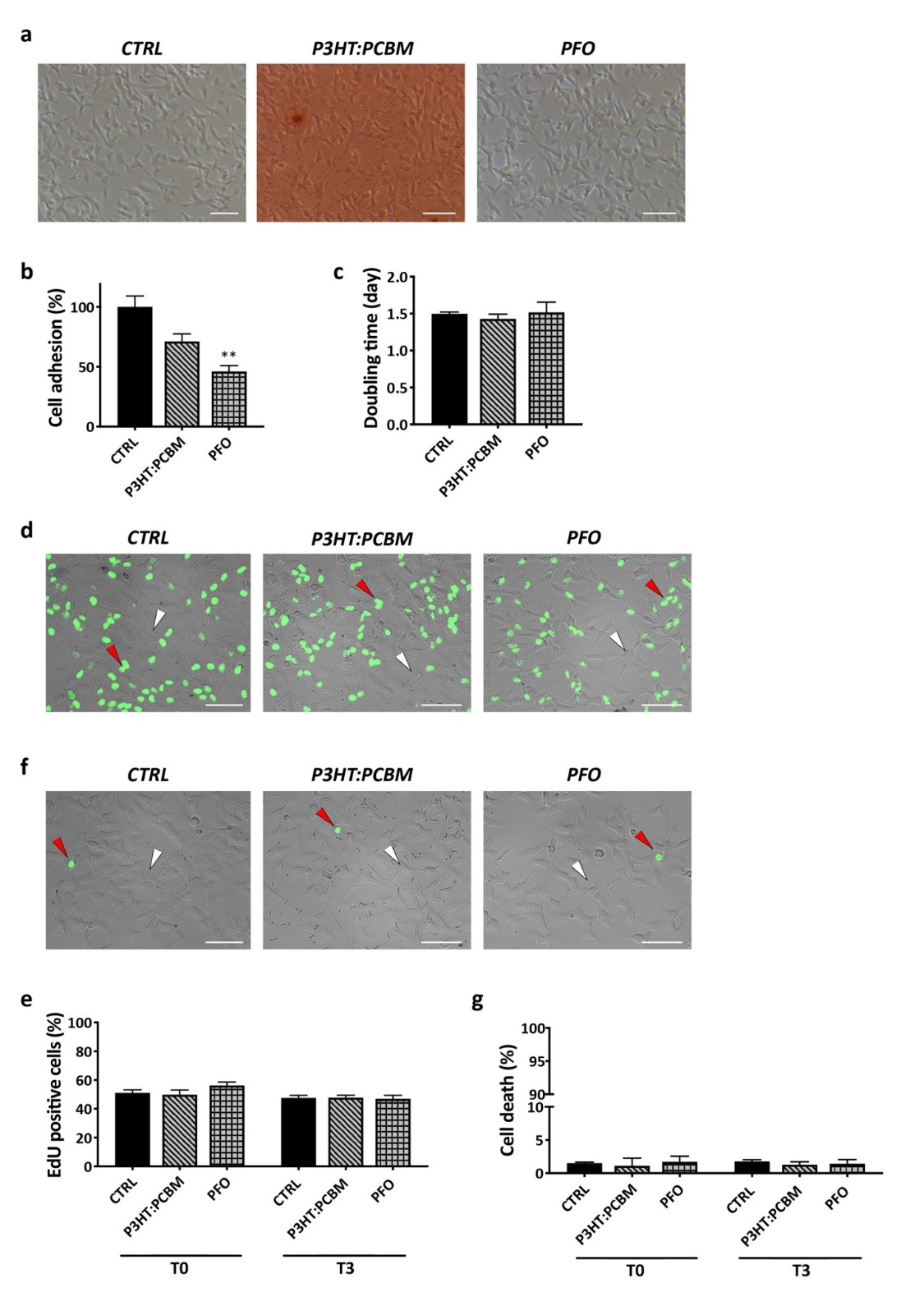


**Supplementary Fig. 6:** **Polymers biocompatibility evaluated as cell adhesion, proliferation and viability. a) Phase contrast micrographs of randomly selected fields of SH-SY5Y cells cultured on standard polystyrene dish (CTRL), on Glass|FTO|P3HT:PCBM substrate (P3HT:PCBM) and on Glass|FTO|PFO substrate (PFO) for three days. Scale bar = 50 μm. b) Cell adhesion evaluated at 16 hours (T0) after SH-SY5Y cell seeding on polystyrene dish (CTRL), on Glass|FTO|P3HT:PCBM substrate (P3HT:PCBM) and on Glass|FTO|PFO substrate (PFO). Adhesion rate of SH-SY5Y cells on P3HT:PCBM and PFO at T0 was about 70% and 50% of the control, respectively (CTRL = 100 ± 9.15 %; P3HT:PCBM = 71.19 ±3.64 %; PFO = 46.18±4.79). Data are expressed as percentage of control (CTRL). Statistical difference vs CTRL ** p<0.01 (Data were analysed with GraphPad Prism software V7.0). c) Cell proliferation evaluated after three days of culture and expressed as cell doubling time for SH-SY5Y cultured on polystyrene dish (CTRL), on P3HT:PCBM and on PFO layers (Data were analysed with GraphPad Prism software V7.0). d) Representative micrographs of SH-SY5Y cells cultured on polystyrene dish (CTRL), on Glass|FTO|P3HT:PCBM substrate (P3HT:PCBM) and on Glass|FTO|PFO substrate (PFO) and analysed via the Click-iT EdU proliferation assay after three days of culture (T3). White and red arrowheads indicate non-proliferating and proliferating (EdU positive) cells, respectively. Scale bar = 50 μm. e) Quantification of cell proliferation measured by Click-iT EdU proliferation assay, expressed as percentage of positive cells on the total number of cells analysed at the beginning (T0) and at the end (T3) of culture (Image J software 1.49V was used. Data were analysed with GraphPad Prism software V7.0). f) Representative micrographs of SH-SY5Y cells cultured on polystyrene dish (CTRL), on Glass|FTO|P3HT:PCBM substrate (P3HT:PCBM) and on Glass|FTO|PFO substrate (PFO) and analysed via the in-situ Cell Viability Imaging assay after three days of culture (T3). White and red arrowheads indicate live and dead (green) cells, respectively. Scale bar = 50 μm. g) Quantification of cell death measured by in-situ Cell Viability Imaging assay, expressed as percentage of dead (green) cells on the total number of cells analysed at the beginning (T0) and at the end (T3) of culture (Image J software 1.49V was used. Data were analysed with GraphPad Prism software V7.0).**


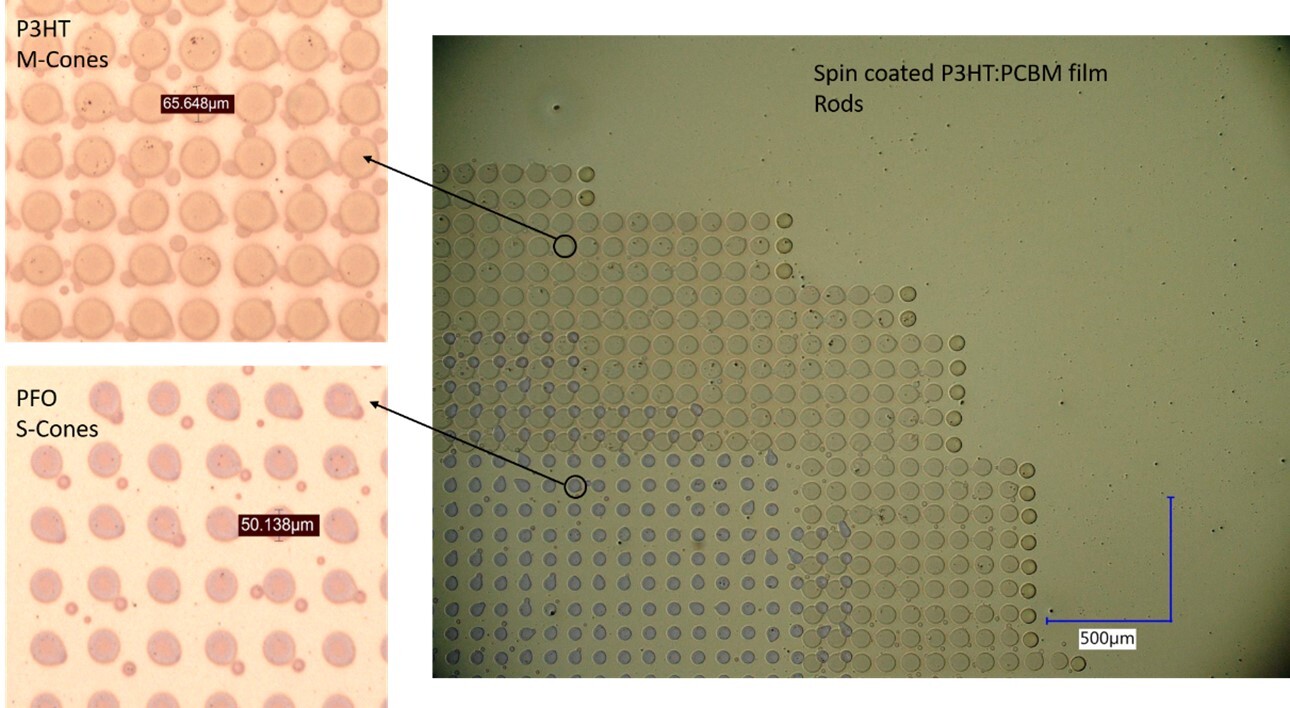


**Supplementary Fig. 7: Optical microscopy images of inkjet-printed photoreceptors-rounded polymer pixels on spin coated polymer thin film. P3HT:PCBM polymer thin film was spin coated on FTO glass substrates (2000rpm, 60 s); P3HT and PFO pixels were printed on P3HT:PCBM film. P3HT pixels diameter was around 65 µm. PFO pixels diameter was around 50 µm.**


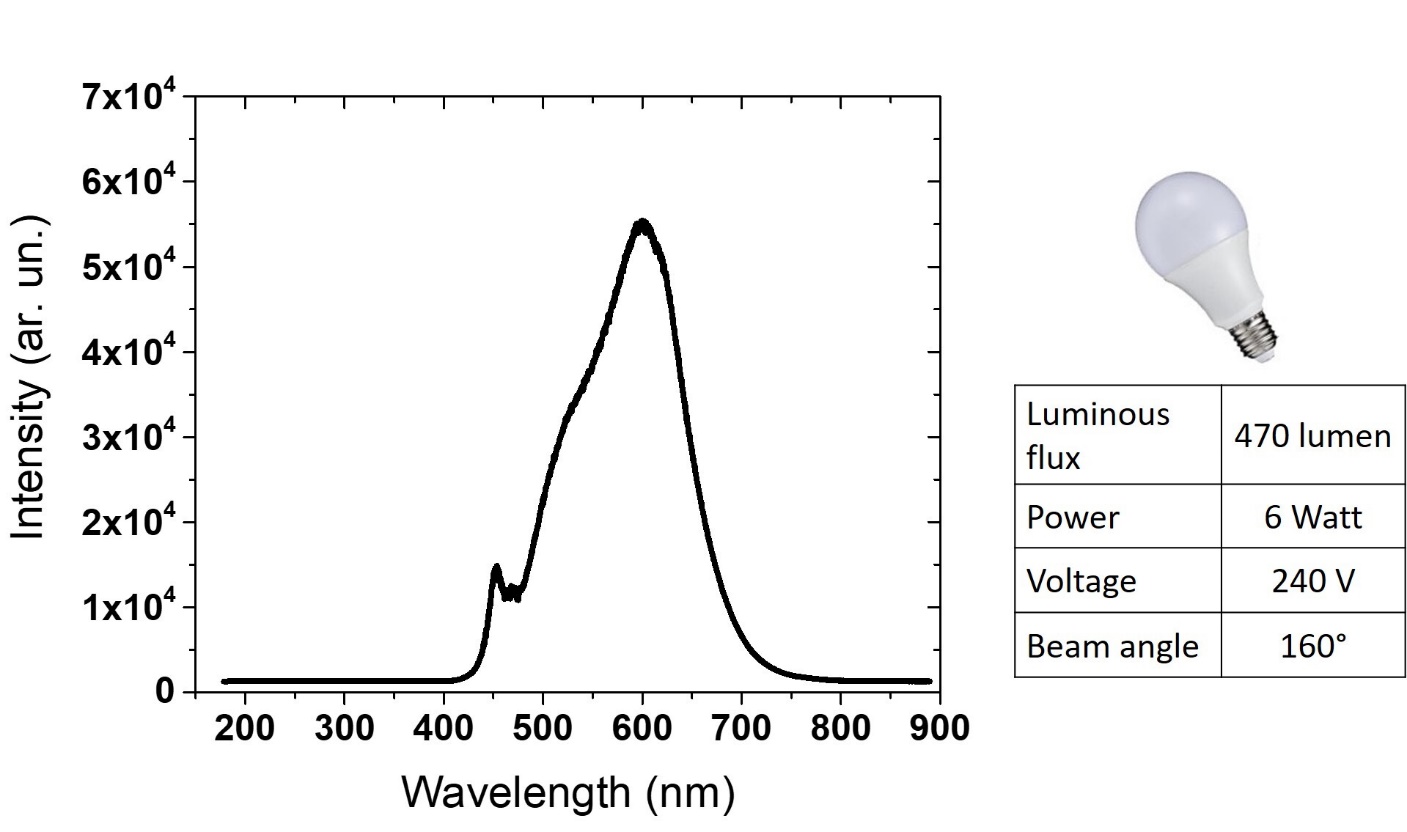


**Supplementary Fig. 8: 6 Watt LED Lamp spectra and characteristics. Standard warm white light shows a spectral range from 500 nm to 700 nm. White light was used as light stimulation. (OriginPro 2016 was used)**


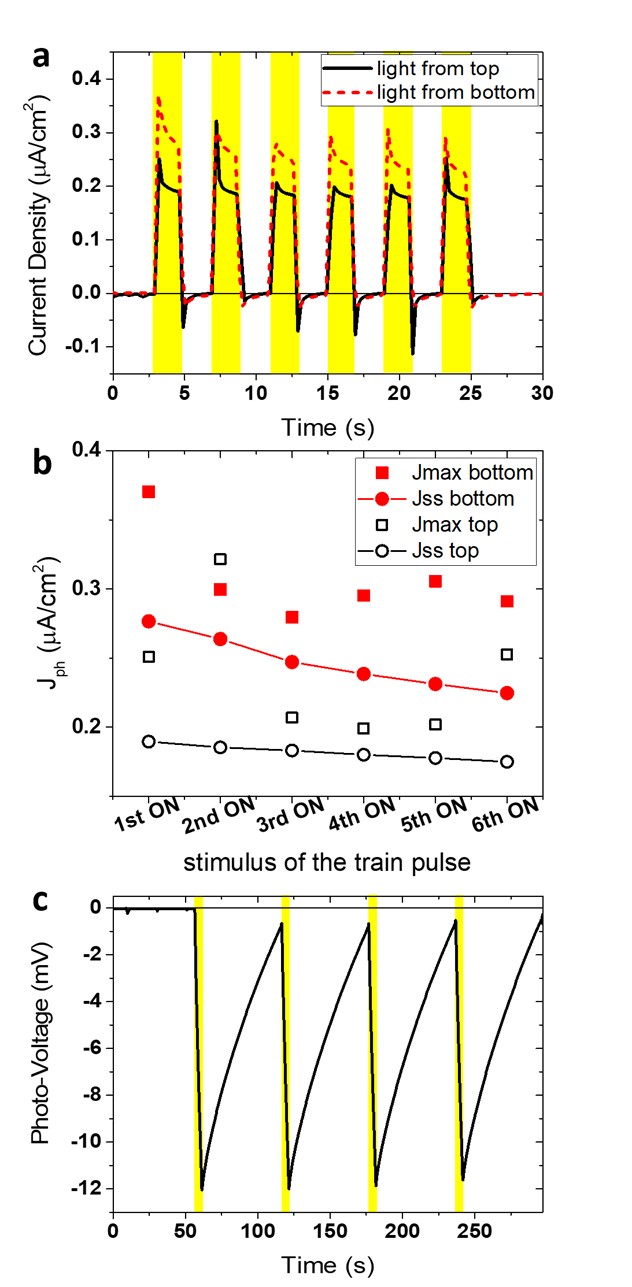


**Supplementary Fig. 9: Opto-electrical outputs of the inkjet-printed artificial retina. a) Current density recorded from the artificial retina device by illuminating it with a train of 6 white light pulses coming from above (black line) or from the bottom (dashed red line). The shaded yellow areas represent the duration of the light stimuli. b) Current Density values for Jmax (maximum value reached after light ON) and Jss (minimum value of the sustained current under light stimulation) by illuminating the device via white LED train of 6 light pulses coming from above (black) and from the bottom (red). c) Vph signals generated from the polymer artificial retina-device when illuminated from above via a train of 6 white light pulses of 5 s light duration alternated with 60 s dark period. (OriginPro 2016 was used)**


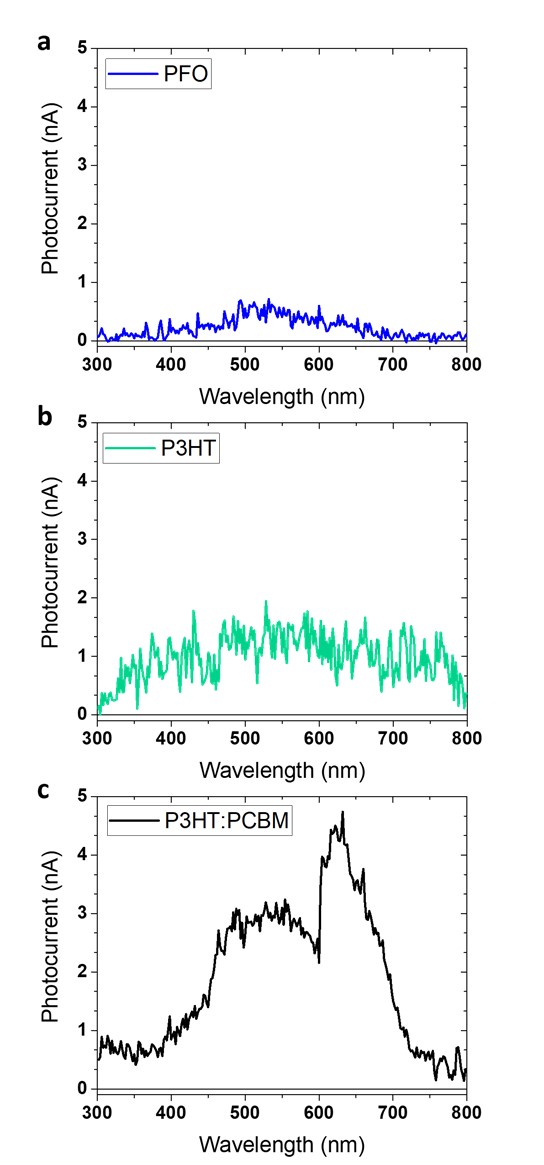


**Supplementary Fig. 10: Photocurrent measurements obtained by illuminating singular polymer annuli of the inkjet-printed retina. a) PFO; b) P3HT; c) P3HT:PCBM. To measure I_ph_ signals coming from each different polymer annulus, customized masks were used in order to illuminate singularly the polymer annulus of interest and masking the leftover printed artificial retina area. (OriginPro 2016 was used)**


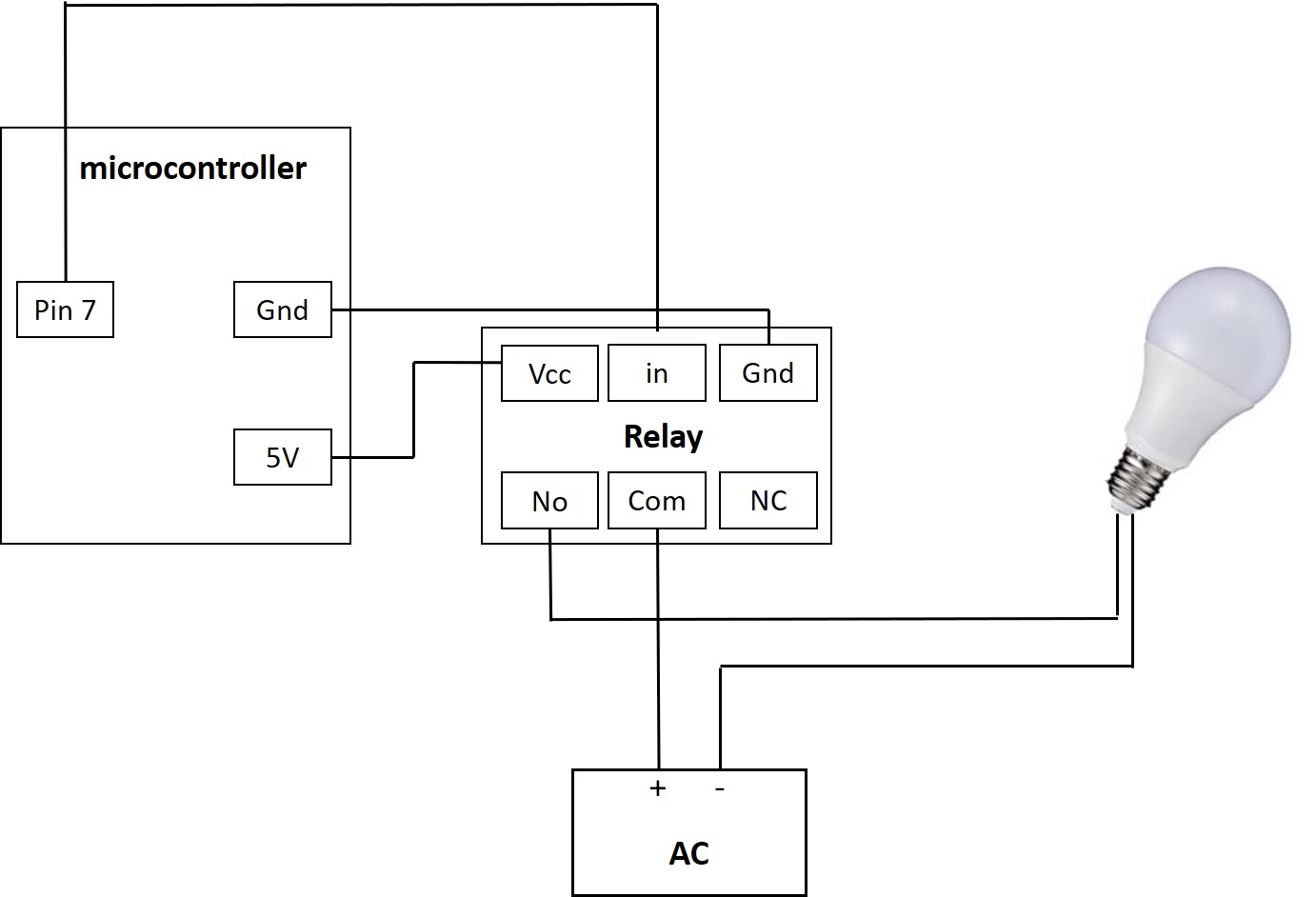


**Supplementary Fig. 11: Microcontroller connections schematics with the 6 W LED Lamp source to obtain light train pulse stimuli alternating 2 s ON and 2 s OFF light periods or 5 s ON and 60 s OFF light period, according the experimental needs. Used microcontroller is ARDUINO-UNO.**
